# Construction and evaluation of a practical model for measuring health-adjusted life expectancy (HALE) in China

**DOI:** 10.1186/s12889-024-19112-6

**Published:** 2024-06-22

**Authors:** San Qian Chen, Yu Cao, Jing Jie Ma, Xing Chao Zhang, Song Bo Hu

**Affiliations:** 1 School of Public Health, Jiangxi Medical College, Nanchang University, Donghu Campus, Nanchang, Jiangxi Province 330006 PR China; 2 Jiangxi Provincial Key Laboratory of Disease Prevention and Public Health, Nanchang University, Nanchang, 330006 PR China

**Keywords:** China, Years lived with disability rate, Health-adjusted life expectancy, Practical model, Uncertainty intervals

## Abstract

**Background:**

HALE is now a regular strategic planning indicator for all levels of the Chinese government. However, HALE measurements necessitate comprehensive data collection and intricate technology. Therefore, effectively converting numerous diseases into the years lived with disability (YLD) rate is a significant challenge for HALE measurements. Our study aimed to construct a simple YLD rate measurement model with high applicability based on the current situation of actual data resources within China to address challenges in measuring HALE target values during planning.

**Methods:**

First, based on the Chinese YLD rate in the Global Burden of Disease (GBD) 2019, Pearson correlation analysis, the global optimum method, etc., was utilized to screen the best predictor variables from the current Chinese data resources. Missing data for predictor variables were filled in via spline interpolation. Then, multiple linear regression models were fitted to construct the YLD rate measurement model. The Sullivan method was used to measure HALE. The Monte Carlo method was employed to generate 95% uncertainty intervals. Finally, model performances were assessed using the mean absolute error (MAE) and mean absolute percentage error (MAPE).

**Results:**

A three-input-parameter model was constructed to measure the age-specific YLD rates by sex in China, directly using the incidence of infectious diseases, the incidence of chronic diseases among persons aged 15 and older, and the addition of an under-five mortality rate covariate. The total MAE and MAPE for the combined YLD rate were 0.0007 and 0.5949%, respectively. The MAE and MAPE of the combined HALE in the 0-year-old group were 0.0341 and 0.0526%, respectively. There were slightly fewer males (0.0197, 0.0311%) than females (0.0501, 0.0755%).

**Conclusion:**

We constructed a high-accuracy model to measure the YLD rate in China by using three monitoring indicators from the Chinese national routine as predictor variables. The model provides a realistic and feasible solution for measuring HALE at the national and especially regional levels, considering limited data.

**Supplementary Information:**

The online version contains supplementary material available at 10.1186/s12889-024-19112-6.

## Background

Economic and social development has shifted individuals’ focus toward not only living longer but also living better. Healthy life expectancy is a significant measure of a population’s lifespan and quality of life [1]. In 2000, the World Health Organization (WHO) explicitly included healthy life expectancy as a health system performance evaluation indicator to quantify the health status of populations [2]. The WHO has since routinely published healthy life expectancy surveillance values for countries worldwide. In 2004, the European Union adopted the healthy life expectancy as a structural indicator for monitoring health trends [3]. France, the United Kingdom, and Sweden even made this a policy goal prior to the European Union. The first planning goal of the “Healthy China 2030” Plan [4], released by the Chinese Communist Party State Council in 2016, states that “by 2030, healthy life expectancy will be significantly improved”.

healthy life expectancy is typically measured following the Sullivan methodology, which combines unhealthy status within a life table framework [5]. With the diversity of health concepts, unhealthy health conditions with different meanings correspond to different healthy life expectancies such as disability-free life expectancy [6], healthy life expectancy without specific diseases [7], and self-rated healthy life expectancy [8]. These metrics are easily calculable, and unhealthy rate data are readily available, with numerous corresponding studies. However, since these healthy life expectancies solely concentrate on the number of years of survival with a specific health condition, they fail to offer a comprehensive and integrated assessment of the quality of health survival and are difficult to compare with each other.

However, the healthy life expectancy indicator in China’s national planning is generally a composite indicator that discounts life expectancy by considering various illnesses and disabilities. Currently, this indicator is dominated by health-adjusted life expectancy (HALE) from the Global Burden of Disease (GBD) study led by The Institute for Health Metrics and Evaluation. For instance, GBD 2019 [9] categorizes all illnesses and disabilities into three major groups and 369 causes for comprehensive discounting, and evaluates the HALE of 204 countries or regions worldwide, including China. This provides an essential point of reference for assessing the quality of comprehensive health levels and formulating policies for each country. The GBD HALE was also calculated using the Sullivan method [10]. The unhealthy rate is the years lived with disability (YLD) rate converted from a combination of all the different types of diseases, for which full prevalence data are needed. However, given the scarcity of data and variety of country-specific problems, GBD has devised a complicated process to estimate the YLD rate. In particular, the technical approach represented by DisMod-MR [11] is difficult to generalize and apply globally.

With the development of GBD YLD rate measurement techniques [12–14], there are now other methods of measurement both domestically and internationally. One method involves collecting disease prevalence data through surveys, and calculating YLD rates by combining the disability weights of each illness [15, 16]. However, the questionnaire is not comprehensive and captures data on only a few diseases. Another approach is to extract prevalence rates for various diseases from electronic medical records in hospitals, again combined with disability weights to compute YLD rates [17, 18]. Nevertheless, determining the full prevalence of nonfatal diseases in a specific area using electronic medical records is likely to lead to underreporting. Moreover, as the data are derived from hospitals and utilize techniques such as big data, the applicability of these methods is limited. Additionally, the WHO [19] proposed using the indirect method to calculate YLD rates, assuming that the ratio of YLD rate to years of life lost is the same in the study and reference areas. Its applications exist in Guangdong Province, China [20, 21]. However, the distribution of disease burden may differ significantly among regions, introducing bias into estimation outcomes [22]. These limitations greatly constrain the measurement and applicability of HALEs in China.

In 2022, China’s 14th Five-Year Plan for National Health [23] further proposed the goal of “increasing HALE in the same proportion as expectancy by 2025”. Subgovernments at all levels in China, including provinces and municipalities, have also set regional HALE goals accordingly. Therefore, how to measure the YLD rate in China and its provinces and cities, based on the current status of the actual data in China, has become the most important challenge for HALE measurements in China.

Therefore, this study utilized the GBD technique and database as a basis for identifying the best predictor variables among the current data resources in China to construct a simple model for measuring China’s YLD rate. This approach aims to simplify China’s HALE calculation. In the absence of domestic data resources in China, this provides a practical and feasible solution for measuring national-level HALEs, especially regional-level HALEs, and offers technical assistance for the quantitative evaluation of health policies.

## Methods

### Sources of data

#### The GBD database

The YLD rates and 95% UI, life tables, HALEs, and corresponding sex-specific data for 21 age groups (0, 1–4, 5–9, 10–14,……, 90–94, and 95+) in China for 1990–2019 were obtained from GBD 2019 data from the Global Health Data Exchange query tool (https://vizhub.healthdata.org/gbd-results/).

#### Domestic data available in China

The data for China’s health care system come mainly from disease surveillance, maternal and child health surveillance, cause-of-death surveillance, censuses, sample surveys (e.g., the National Health Service Survey), and residents’ health records, among others. These data are publicly available through vehicles such as the National Health Service Survey and Analysis Report, the China Health and Health Statistics Yearbook, and the China Statistical Yearbook. The National Health Service Survey and Analysis Report offers data on resident prevalence, long-term disabling and disability conditions, etc. The China Health and Health Statistics Yearbook includes data on morbidity and mortality from infectious diseases and maternal and child health (neonatal mortality rate, infant mortality rate, under-five mortality rate (U5MR). The China Statistical Yearbook contains data on national accounting, socioeconomic indicators, educational attainment per capita, and urbanization rate.

### Treatment and screening of independent variables

We filled in missing data for certain variables within China using spline interpolation. Additionally, we harmonized individual indicators with age ranges that were inconsistent across different years.

To screen independent variables, we utilized multiple linear regression, Pearson correlation analysis, collinearity statistics, etc., to screen four variables using the global optimum method. The data included the incidence of infectious diseases (IID), the incidence of chronic diseases among persons aged 15 and older (PCDPF), the two-week incidence of impairment poisoning, and the U5MR. After careful consideration of the model’s applicability, we ultimately selected three independent variables (IID, PCDPF, and U5MR). For reference, the four-input-parameter model has been included in the Appendix, along with additional information. In addition, it should be noted that IID, as referenced in this study, represents the incidence of A and B statutory infectious diseases. See also the Appendix for specific classifications.

### Model construction

#### Modeling of YLD rates by sex and age in China

The model was developed by inputting 3 predictor variables (IID, PCDPF, U5MR) and outputting them to obtain 21 age-specific YLD rates. The details are as follows:

(1) Twenty-one multiple linear regression models were constructed:

1 $${\Gamma }\left({\text{Y}}_{i}\right)={\beta }_{i}+{a}_{i}{X}_{1}+{b}_{i}{X}_{2}+{c}_{i}{X}_{3}$$

In this model, ${\text{Y}}_{i}$ represents the YLD rate for 21 age groups (age Group 0, 1–4, 5–9, 10–14,…, 90–94, and 95+); ${X}_{1}$, ${X}_{2}$, and ${X}_{3}$ correspond to IID, PCDPF, and U5MR, respectively; ${\beta }_{i}$ is the intercept; and ${a}_{i}$, ${b}_{i}$, and ${c}_{i}$ are the corresponding independent variable regression coefficients. Model (1) included 21 multiple linear regression models corresponding to each age group. After testing different transformations, ${\Gamma }\left({\text{Y}}_{i}\right)$ chooses the logit transformation:

2 $${\Gamma }\left({\text{Y}}_{i}\right)=0.5\text{ln}\left(\frac{1-{\text{Y}}_{i}}{{\text{Y}}_{i}}\right)$$

(2) In building Model (1) for different genders, except for the PCDPF, the two independent variables are directly modeled by substituting the aggregate rate for the subgender rate.

#### Sullivan method for measuring HALE[24]

### Uncertainty intervals (UIs)

We assessed the uncertainty of the model coefficients, YLD rates and HALE estimates based on the uncertainty of the GBD data. Monte Carlo methods were utilized to generate 95% UIs. The GBD YLD rate, following logit transformation, was assumed to conform to a multivariate normal distribution. With the GBD-reported YLD rates and 95% UIs, we constructed 30 multivariate normal distributions from which we randomly generated 1000 ${\Gamma }\left({\text{Y}}_{i}\right)$s. Then, we obtained the 95% UIs for the YLD rate estimates, HALE estimates, ${\beta }_{i}$, ${a}_{i}$, ${b}_{i}$, and ${c}_{i}$ using the 2.5th and 97.5th quartiles. More details are given in the Appendix.

### Error assessment

This study evaluated the accuracy of this simple model on the basis of the YLD rate and HALE dimensions. The model-measured YLD and HALE were used as model fit values. HALEs were calculated using model-measured YLD rates and GBD life tables through the Sullivan method. The YLD rate and HALE published by GDB served as true reference values. The data were assessed in three ways: (a) Drawing residual plots. (b) The mean absolute error (MAE) and mean absolute percentage error (MAPE) were calculated. The formulas are as follows [25], where ${\widehat{y}}_{i}$, ${y}_{i}$, and $n$ denote the model fitted value, the true reference value, and the total number of estimates, respectively. (c) Plotting line plots of model-fitted and true reference values with 95% UI.

3 $$MAE=\frac{1}{n}\sum _{i=1}^{n}\left|{\widehat{y}}_{i}-{y}_{i}\right|$$

4 $$MAPE=\frac{100\%}{n}\sum _{i=1}^{n}\left|\frac{{\widehat{y}}_{i}-{y}_{i}}{{y}_{i}}\right|$$

### Statistical software

R-4.2.2 software (packages reshape2, MASS, splines, pracma, gplot2, gcookbook, dplyr, gpubr, gsci) was used throughout this study.

## Results

### Model construction

#### Descriptive analysis of three predictor variables

Table 1 shows the descriptive analysis of the three predictor variables IID, PCDPF (both male and female populations), and U5MR using 4 metrics: maximum, minimum, median, and mean.

**Table 1:** Descriptive analysis of three predictor variables

variables	maximum	minimum	median	mean
IID	0.0030	0.0017	0.0022	0.0023
PCDPF_both	0.5371	0.1862	0.2043	0.2656
PCDPF_male	0.5289	0.1678	0.1864	0.2463
PCDPF_female	0.5423	0.2057	0.2250	0.2846
U5MR	0.0610	0.0078	0.0238	0.0289

#### Coefficients of the YLD rate measurement models

Table 2 displays the coefficients and their 95% UIs for Model (1), the YLD rate measurement model, across various genders and age groups. To ensure accurate model parameters, the regression coefficients were rounded to four decimal places. For age-specific models, all ${\beta }_{i}$ values are positive, while ${a}_{i}$, ${b}_{i}$, and ${c}_{i}$ values may be positive or negative. The ${\beta }_{i}$, ${a}_{i}$, ${b}_{i}$, and ${c}_{i}$ values of the combined model are situated between the corresponding values of the male and female models. After all the coefficients are substituted into Model (1), only three independent variables—IID, PCDPF, and U5MR—are needed to calculate the 21 age-specific YLD rates for males, females, and the combined population in a region. Additionally, the results of the four-parameter model can be found in the Appendix.

### Error assessment

#### Results of YLD rate error assessment

**Fig. 1:**
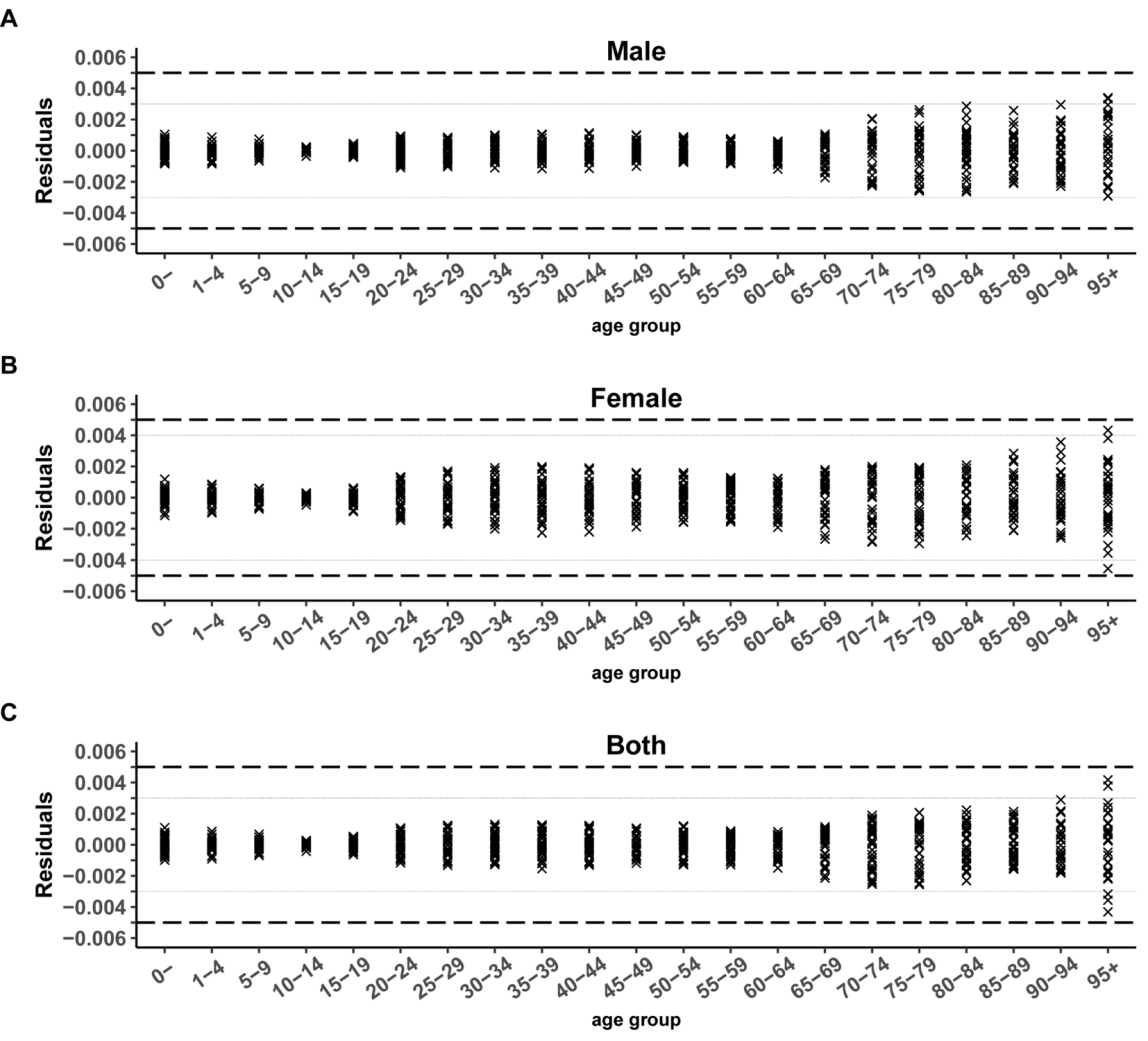
Residuals of YLD rates by genders and age groups

Figure 1 shows the YLD rates for different sexes and age groups. The residuals for males, females, and the combined group fluctuated above and below zero, ranging from (-0.003, 0.003), (-0.004, 0.004), and (-0.003, 0.003), respectively. Table 3 displays the error assessment results for YLD rates across sexes and age groups, with a total MAE and MAPE of 0.0007 and 0.5949%, respectively, for the combined YLD rates. The total MAE and MAPE for males were 0.0006 and 0.5522%, respectively, which were lower than those for females (0.0008 and 0.6762%, respectively). The MAPEs were slightly lower in males than in females in all age groups, except for those aged 0 years. Among the various age groups, there was a general trend of increasing MAE with age, and the MAPE exhibited a general decreasing trend with age.

**Fig. 2:**
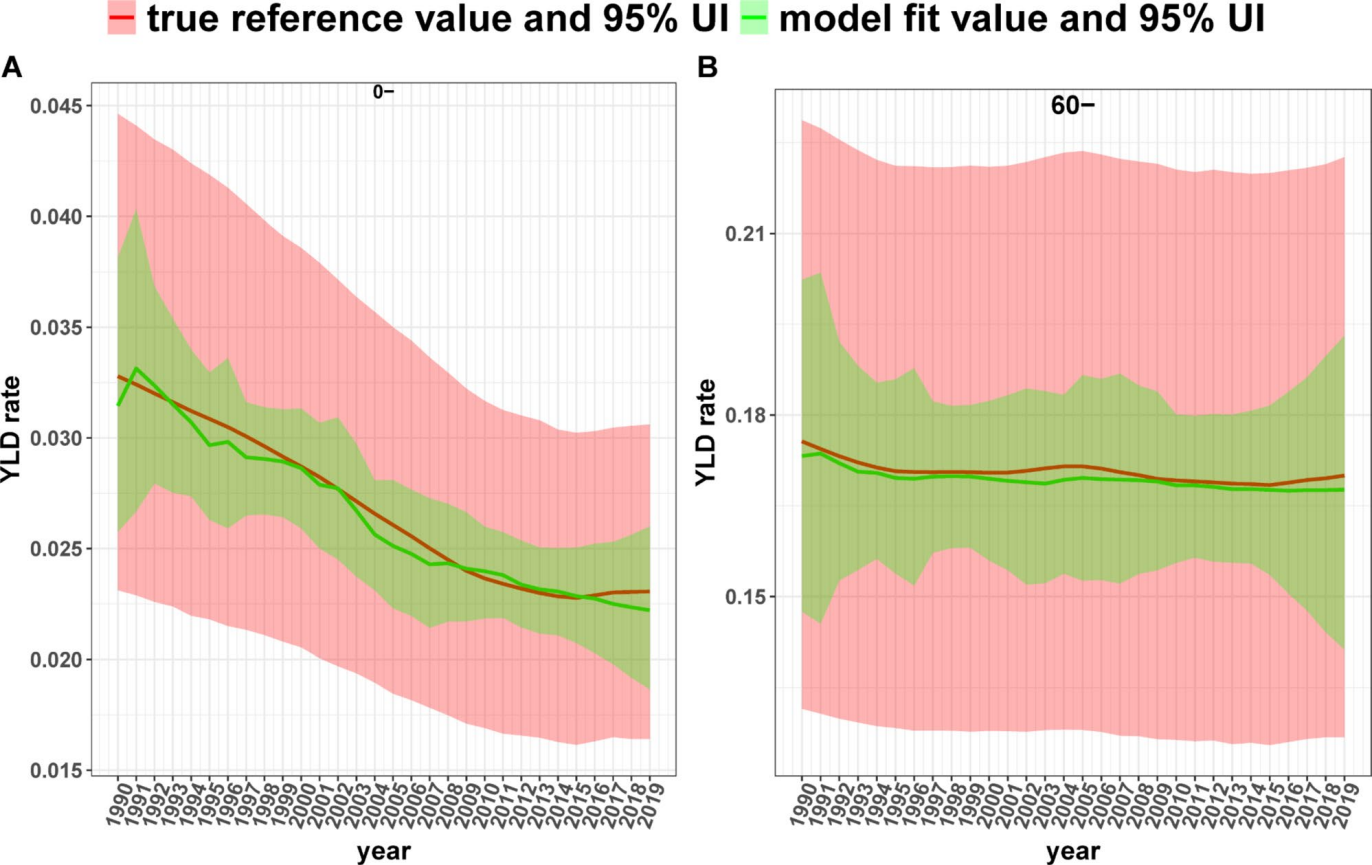
Comparison of model-fitted values and true reference values of the combined YLD rate for different age groups, 1990–2019 (**A**) and (**B**) indicate the 0- age group and 60–64 age group separately. Shaded areas indicate the corresponding 95% uncertainty intervals

In addition, Fig. 2 illustrates the model fit and true reference values of the combined YLD rates for different years for the 0- and 60–64 age groups. Notably, the two lines almost overlap. Comparable observations are observed in other age groups (see Figure S4 in the Appendix).

**Table 2:** Coefficients of the YLD rate measurement model for different genders and age groups

Age groups	Male				Female				Both			
	${\beta }_{i}$	${a}_{i}$	${b}_{i}$	${c}_{i}$	${\beta }_{i}$	${a}_{i}$	${b}_{i}$	${c}_{i}$	${\beta }_{i}$	${a}_{i}$	${b}_{i}$	${c}_{i}$
0-	1.8615(1.5676,2.1686)	8.9296(-89.86,104.4651)	0.0749(-0.3495,0.5163)	-3.4638(-6.2056,-0.6767)	1.9244(1.6017,2.2606)	1.4015(-89.2012,98.5111)	-0.0339(-0.5089,0.4189)	-4.0875(-6.7341,-0.9566)	1.8893(1.5782,2.2044)	5.0838(-95.0983,98.3153)	0.0277(-0.3935,0.4767)	-3.7456(-6.4152,-0.8885)
1–4	1.9694(1.6564,2.282)	-5.1199(-105.66,89.3598)	-0.2137(-0.6211,0.2179)	-2.7403(-5.311,0.1668)	2.0788(1.7471,2.4175)	-9.8239(-103.8426,90.4263)	-0.3049(-0.7628,0.1747)	-3.5109(-6.3764,-0.5827)	2.0151(1.6647,2.3346)	-6.7746(-108.6964,93.8619)	-0.2498(-0.6564,0.2006)	-3.0582(-5.7274,-0.1229)
5–9	1.8413(1.525,2.1515)	-0.8975(-101.7561,94.1937)	-0.1547(-0.569,0.2849)	-1.9308(-4.5082,1.0019)	1.9073(1.5992,2.2224)	-0.0878(-89.1423,95.2822)	-0.1897(-0.6449,0.2733)	-2.3390(-5.0928,0.4604)	1.8688(1.5419,2.19)	-0.1318(-97.964,99.0181)	-0.1682(-0.5808,0.2811)	-2.0884(-4.6995,0.8061)
10–14	1.6316(1.3374,1.9374)	1.9377(-97.2023,95.3694)	-0.0537(-0.4723,0.3893)	-0.8173(-3.4299,2.0455)	1.5875(1.2654,1.9069)	1.5146(-83.0149,99.3105)	-0.0599(-0.5111,0.391)	-0.7935(-3.4908,1.9591)	1.6099(1.2935,1.9257)	1.9764(-90.8264,97.9303)	-0.0552(-0.4808,0.3775)	-0.8259(-3.3644,1.7507)
15–19	1.5261(1.2449,1.8354)	3.7940(-93.8766,94.1744)	-0.0335(-0.4508,0.3749)	-0.6617(-3.3066,2.0369)	1.4563(1.1364,1.7867)	-1.4657(-92.6654,94.5287)	-0.0635(-0.5362,0.4028)	-1.2921(-4.0064,1.5633)	1.4898(1.171,1.7968)	0.9114(-91.2431,95.0335)	-0.0420(-0.4779,0.4019)	-1.0058(-3.4936,1.5461)
20–24	1.4536(1.1914,1.738)	4.2732(-82.274,88.3974)	-0.0374(-0.4256,0.3432)	-1.2270(-3.6582,1.2982)	1.3738(1.0737,1.6944)	-2.7204(-89.8365,88.28)	-0.0654(-0.5239,0.3675)	-2.0357(-4.5894,0.7386)	1.4092(1.115,1.698)	0.2690(-86.5912,85.2794)	-0.0420(-0.4448,0.3843)	-1.6123(-4.0458,0.7989)
25–29	1.3981(1.145,1.6852)	-0.6930(-92.0414,85.5352)	-0.0710(-0.4481,0.3159)	-1.2683(-3.7395,1.239)	1.2745(0.9582,1.6023)	-3.5984(-95.8204,89.5813)	-0.0426(-0.5183,0.4044)	-1.9072(-4.5349,1.0659)	1.3340(1.0227,1.6283)	-3.0808(-92.6824,84.617)	-0.0532(-0.4668,0.3865)	-1.5637(-4.0372,0.9158)
30–34	1.3550(1.0825,1.6537)	-3.8105(-99.3002,86.6227)	-0.0995(-0.5006,0.292)	-1.2241(-3.7556,1.476)	1.1988(0.8692,1.5326)	-3.6009(-98.9042,89.4311)	-0.0307(-0.5154,0.4211)	-1.7896(-4.459,1.2694)	1.2725(0.9508,1.5746)	-3.3793(-97.621,90.2346)	-0.0623(-0.489,0.3835)	-1.4924(-4.0047,1.1893)
35–39	1.3017(1.0351,1.5927)	-4.8401(-99.3466,81.1911)	-0.1094(-0.4948,0.2805)	-1.2015(-3.6518,1.3679)	1.1471(0.8328,1.4698)	-5.9297(-98.9376,87.4883)	-0.0362(-0.5058,0.4076)	-1.7220(-4.3229,1.2322)	1.2219(0.9178,1.5141)	-5.4039(-94.3819,84.6567)	-0.0707(-0.4856,0.3752)	-1.4605(-3.9564,1.1055)
40–44	1.2235(0.9435,1.5236)	-3.9177(-98.8668,84.7891)	-0.1054(-0.4978,0.3085)	-0.9989(-3.4551,1.6916)	1.0765(0.7607,1.4012)	-6.3659(-101.6396,87.6795)	-0.0302(-0.5097,0.4269)	-1.4290(-4.0477,1.5179)	1.1470(0.8339,1.4453)	-4.9543(-98.126,85.1809)	-0.0656(-0.4943,0.3699)	-1.1952(-3.738,1.4064)
45–49	1.1470(0.8608,1.4537)	-4.3704(-101.06,85.1792)	-0.0855(-0.4893,0.3345)	-0.8279(-3.3536,1.938)	1.0049(0.6872,1.3301)	-5.0810(-99.3075,88.5691)	-0.0269(-0.5162,0.4319)	-1.1509(-3.7588,1.7713)	1.0716(0.7573,1.3766)	-3.9819(-98.1502,89.3272)	-0.0542(-0.4911,0.4001)	-0.9499(-3.5559,1.6995)
50–54	1.0640(0.7779,1.3822)	-5.0987(-101.6711,86.558)	-0.0629(-0.4766,0.3672)	-0.6543(-3.2642,2.1167)	0.9263(0.6099,1.2474)	-5.3515(-98.0374,89.4413)	-0.0176(-0.5021,0.4305)	-0.9582(-3.5471,1.9832)	0.9918(0.6765,1.2982)	-4.8706(-101.1156,87.1881)	-0.0399(-0.4833,0.4161)	-0.7512(-3.3769,1.9276)
55–59	0.9734(0.6885,1.2923)	-6.1668(-100.2057,90.1042)	-0.0428(-0.458,0.3824)	-0.5263(-3.1987,2.1742)	0.8550(0.5407,1.1749)	-7.3568(-103.2215,83.4189)	-0.0097(-0.4922,0.4641)	-0.7126(-3.3674,2.1542)	0.9136(0.5897,1.2317)	-7.0781(-101.4935,83.6403)	-0.0272(-0.4707,0.4594)	-0.5775(-3.2193,2.072)
60–64	0.8716(0.5849,1.1754)	-7.2787(-103.3529,89.184)	-0.0142(-0.4208,0.416)	-0.3027(-2.9157,2.3396)	0.7680(0.4397,1.0843)	-8.3920(-103.148,87.0003)	0.0077(-0.4696,0.4859)	-0.4027(-3.0952,2.5065)	0.8216(0.5069,1.1228)	-8.2262(-100.3827,80.3776)	-0.0086(-0.4604,0.4812)	-0.3444(-3.0742,2.4549)
65–69	0.7501(0.4676,1.0324)	-7.0827(-95.9519,79.0654)	0.0298(-0.3817,0.4741)	-0.1241(-2.8241,2.5645)	0.6620(0.3442,0.9885)	-7.7256(-101.1817,90.0398)	0.0263(-0.4443,0.4832)	-0.2158(-2.9427,2.6614)	0.7091(0.4118,1.001)	-7.7619(-94.8636,77.8574)	0.0199(-0.4051,0.4821)	-0.2119(-2.8057,2.4425)
70–74	0.6373(0.3512,0.9403)	-6.3963(-97.9173,82.0546)	0.0507(-0.346,0.4975)	0.0401(-2.8468,2.8818)	0.5604(0.2521,0.8764)	-6.6255(-97.2704,91.4211)	0.0292(-0.4452,0.4706)	-0.0710(-2.7701,2.7855)	0.6016(0.3059,0.9141)	-6.9564(-99.3681,76.6746)	0.0330(-0.3856,0.4962)	-0.0923(-2.7044,2.6144)
75–79	0.5494(0.2213,0.8751)	-5.3748(-105.2794,88.8814)	0.0363(-0.4093,0.4931)	0.2235(-2.8412,3.175)	0.4713(0.1574,0.7996)	-5.5550(-101.808,90.0585)	0.0143(-0.4464,0.4868)	0.0803(-2.7465,2.8094)	0.5094(0.1977,0.8408)	-5.5586(-97.4039,87.087)	0.0228(-0.4503,0.4946)	0.0533(-2.5135,2.9136)
80–84	0.4613(0.1199,0.7939)	-3.3880(-101.8622,102.616)	0.0117(-0.4504,0.4671)	0.4306(-2.4492,3.3016)	0.3734(0.0518,0.6938)	-2.7735(-95.5256,93.9542)	-0.0082(-0.4921,0.4961)	0.2355(-2.7983,3.0682)	0.4092(0.0962,0.7623)	-2.8768(-99.8572,93.4903)	0.0049(-0.4803,0.4952)	0.2370(-2.6374,3.2957)
85–89	0.3952(0.0485,0.7345)	-0.6385(-102.0297,112.085)	-0.0151(-0.4789,0.4497)	0.4766(-2.5766,3.5764)	0.3067(-0.0217,0.6456)	1.3770(-99.3191,99.616)	-0.0421(-0.5589,0.4754)	0.0682(-2.9729,3.0247)	0.3354(0.0222,0.6869)	0.0632(-100.3999,98.5852)	-0.0277(-0.5356,0.4512)	0.1716(-3.0417,3.2445)
90–94	0.3365(-0.0128,0.6713)	3.5145(-96.6604,114.9733)	-0.0338(-0.5164,0.4335)	0.6014(-2.3655,3.638)	0.2523(-0.0798,0.5816)	6.5459(-87.0686,108.0101)	-0.0812(-0.615,0.4209)	-0.1256(-3.2322,2.9552)	0.2702(-0.0434,0.5961)	5.1485(-93.6325,106.3527)	-0.0843(-0.5841,0.3881)	0.0399(-3.0837,3.047)
95+	0.2630(-0.0767,0.5999)	8.7173(-92.0022,121.9523)	-0.0469(-0.5513,0.4205)	0.6455(-2.3148,3.64)	0.1828(-0.1644,0.5225)	12.5105(-86.6712,113.2924)	-0.1043(-0.6223,0.3976)	-0.3791(-3.5573,2.5804)	0.1937(-0.1699,0.5055)	12.2797(-87.3525,121.4148)	-0.1106(-0.6319,0.3815)	-0.2437(-3.0914,2.9493)

Values in parentheses are 95% uncertainty intervals

**Table 3:** Results of error assessment of YLD rates by genders and age groups

age groups	Male		Female		Both	
	MAE	MAPE	MAE	MAPE	MAE	MAPE
0-	0.0005	1.7540	0.0004	1.5975	0.0004	1.6616
1–4	0.0003	0.9893	0.0003	1.3444	0.0003	1.1323
5–9	0.0002	0.7038	0.0002	0.8305	0.0002	0.7539
10–14	0.0001	0.2634	0.0002	0.3706	0.0001	0.3190
15–19	0.0002	0.3781	0.0003	0.5477	0.0002	0.4717
20–24	0.0005	0.8592	0.0007	0.9664	0.0006	0.8944
25–29	0.0005	0.7652	0.0008	1.0014	0.0006	0.8637
30–34	0.0005	0.6411	0.0009	0.9424	0.0006	0.7739
35–39	0.0005	0.5793	0.0009	0.8863	0.0006	0.6839
40–44	0.0005	0.5437	0.0009	0.7656	0.0006	0.6220
45–49	0.0004	0.4308	0.0008	0.6231	0.0005	0.4329
50–54	0.0004	0.3567	0.0008	0.5509	0.0006	0.4418
55–59	0.0004	0.2952	0.0008	0.4699	0.0006	0.3736
60–64	0.0004	0.2325	0.0008	0.4186	0.0005	0.3110
65–69	0.0007	0.3499	0.0011	0.4924	0.0008	0.4154
70–74	0.0011	0.4852	0.0013	0.5373	0.0012	0.5097
75–79	0.0012	0.4643	0.0014	0.4795	0.0012	0.4607
80–84	0.0011	0.3956	0.0012	0.3591	0.0012	0.3951
85–89	0.0009	0.2834	0.0011	0.3086	0.0010	0.2918
90–94	0.0012	0.3509	0.0012	0.3190	0.0010	0.2775
95+	0.0017	0.4751	0.0016	0.3896	0.0017	0.4076
Total	0.0006	0.5522	0.0008	0.6762	0.0007	0.5949

mean absolute error (MAE), mean absolute percentage error (MAPE), MAE is in units of 1 and MAPE is in %

**Table 4:** Results of error assessment of HALE by genders and age groups

age groups	Male		Female		Both	
	MAE	MAPE	MAE	MAPE	MAE	MAPE
0-	0.0197	0.0311	0.0501	0.0755	0.0341	0.0526
1–4	0.0200	0.0313	0.0508	0.0762	0.0346	0.0529
5–9	0.0194	0.0321	0.0504	0.0797	0.0341	0.0551
10–14	0.0189	0.0339	0.0498	0.0852	0.0337	0.0590
15–19	0.0187	0.0365	0.0493	0.0917	0.0333	0.0635
20–24	0.0180	0.0386	0.0480	0.0976	0.0323	0.0675
25–29	0.0163	0.0388	0.0450	0.1008	0.0301	0.0694
30–34	0.0147	0.0391	0.0414	0.1030	0.0272	0.0699
35–39	0.0134	0.0403	0.0381	0.1063	0.0248	0.0720
40–44	0.0125	0.0429	0.0345	0.1094	0.0227	0.0752
45–49	0.0127	0.0507	0.0310	0.1132	0.0212	0.0814
50–54	0.0126	0.0602	0.0280	0.1202	0.0199	0.0902
55–59	0.0128	0.0739	0.0252	0.1295	0.0184	0.1003
60–64	0.0126	0.0912	0.0226	0.1433	0.0170	0.1154
65–69	0.0123	0.1155	0.0199	0.1609	0.0160	0.1391
70–74	0.0111	0.1410	0.0162	0.1733	0.0136	0.1575
75–79	0.0084	0.1467	0.0122	0.1783	0.0103	0.1636
80–84	0.0057	0.1456	0.0082	0.1705	0.0069	0.1576
85–89	0.0035	0.1352	0.0060	0.1775	0.0047	0.1534
90–94	0.0037	0.1784	0.0050	0.2059	0.0039	0.1683
95+	0.0038	0.2565	0.0045	0.2797	0.0046	0.2871
Total	0.0129	0.0838	0.0303	0.1323	0.0211	0.1072

mean absolute error (MAE), mean absolute percentage error (MAPE), MAE is in units of 1 and MAPE is in %

#### Results of HALE error assessment

**Fig. 3:**
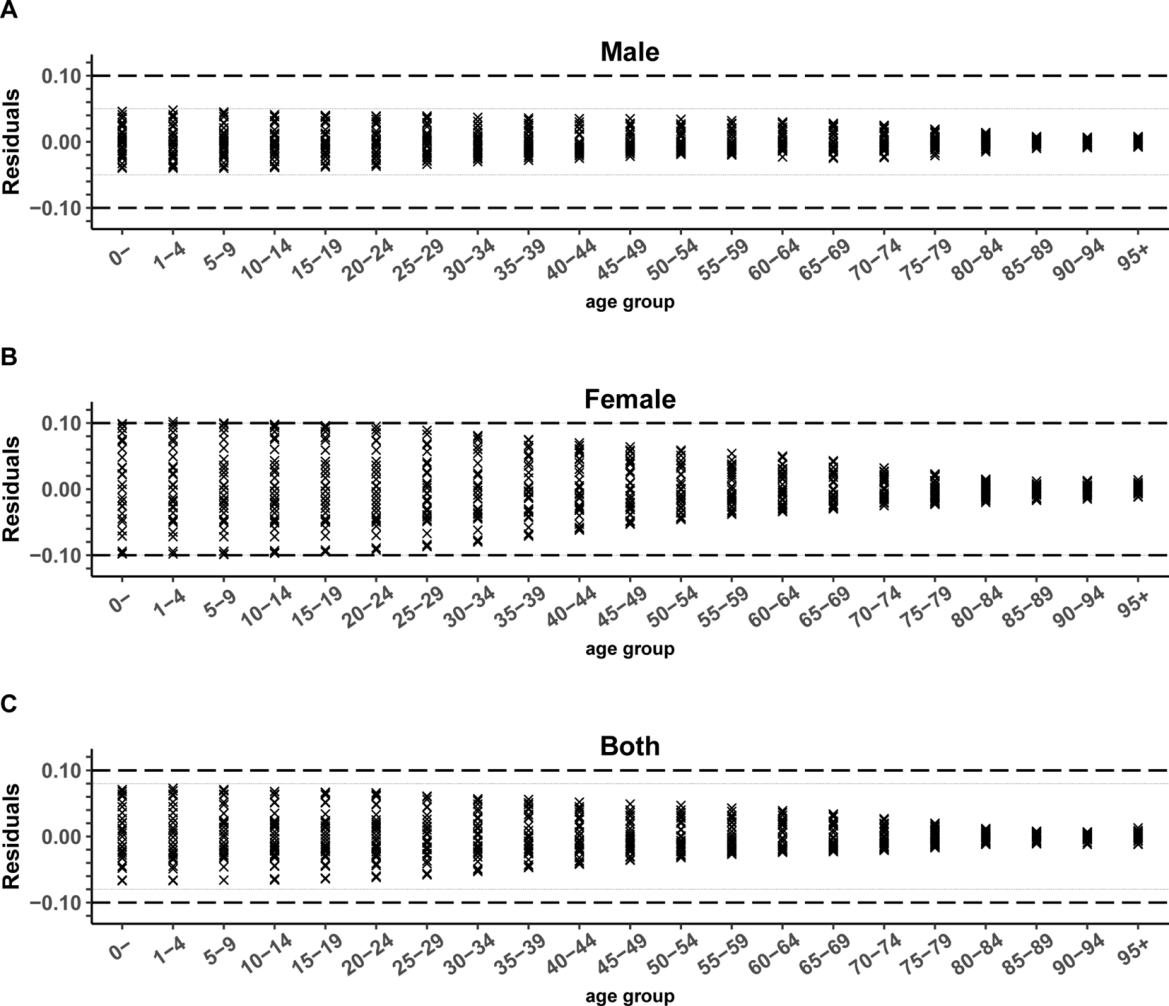
Residuals of HALE by genders and age groups

**Fig. 4:**
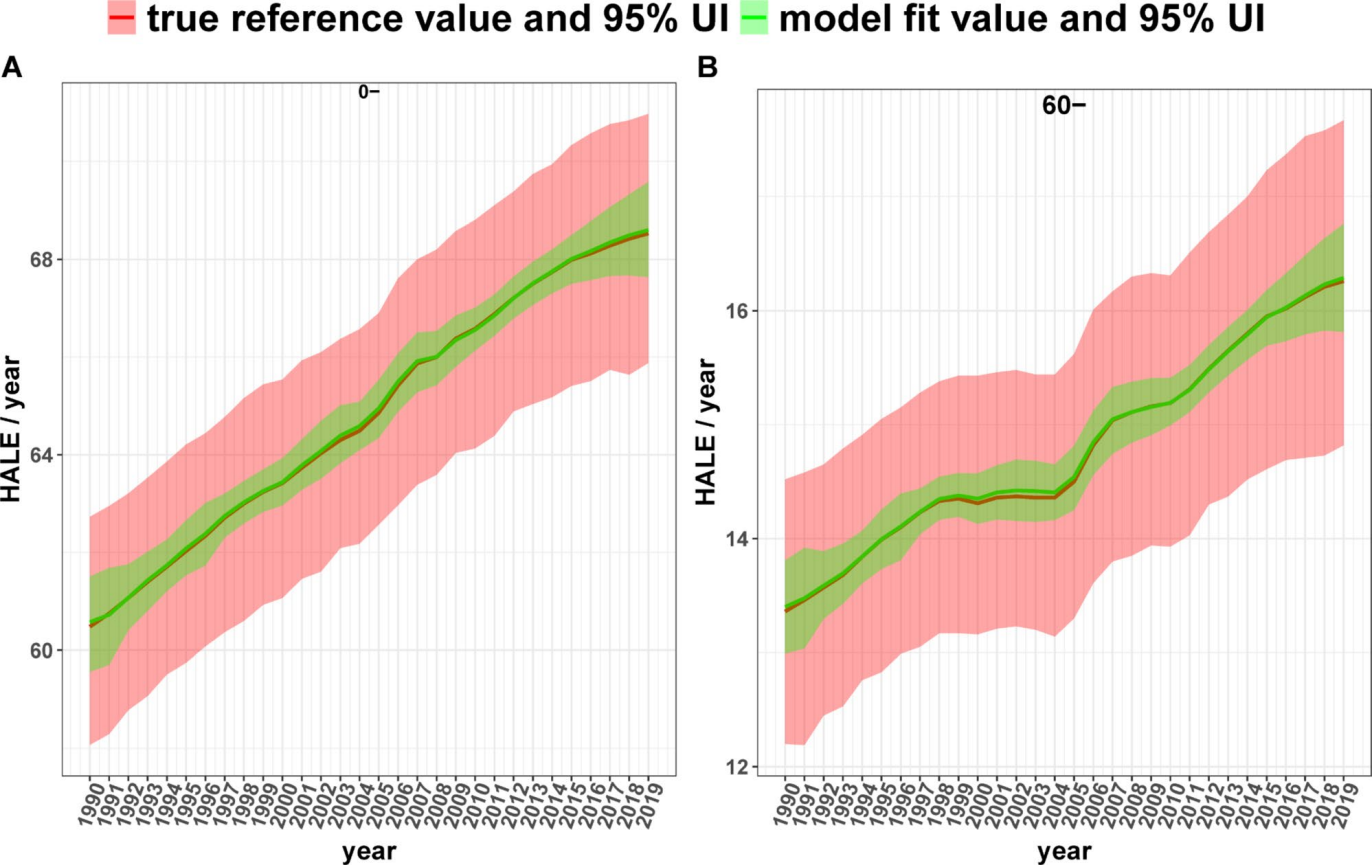
Comparison of model-fitted values and true reference values of the combined HALE for different age groups, 1990–2019 (**A**) and (**B**) indicate the 0- age group and 60–64 age group separately. Shaded areas indicate the corresponding 95% uncertainty intervals

The residuals for males, females, and patients with combined HALE also fluctuated above and below 0, with ranges of (-0.05, 0.05), (-0.10, 0.10), and (-0.08, 0.08), respectively (see Fig. 3). The MAE and MAPE of the combined HALE in the 0-year age group were 0.0341 years and 0.0526%, respectively. These metrics were lower for males (0.0197 years, 0.0311%) than for females (0.0501 years, 0.0755%). In the 60-year-old group, the MAE and MAPE of the combined HALE were 0.0170 and 0.1154%, respectively, and were lower in males (0.0126, 0.0912%) than in females (0.0226, 0.1433%). The male MAPEs were slightly lower than the female MAPEs in all age groups, except for the 95 + years group. Refer to Table 4. Again, the model fit values for the combined HALE and the true reference values nearly overlap. See Fig. 4 and Appendix (Figure S5).

## Discussion

This study employed the GBD Chinese YLD rate as a benchmark to identify three commonly used indicators, IID, PCDPF and U5MR, from available data resources within China. A model for measuring the Chinese YLD rate with these three variables as predictors was then constructed. The model will have significant applications in the measurement of HALE in mainland China.

Since the HALE incorporated into Chinese government planning in the document ‘Healthy China 2030’ in 2016 [4]. It has become standard practice for all levels of government in China, including the entire country, 34 provinces and 333 municipalities, to propose target planning for HALE. Nevertheless, the calculation of HALE in planning is subject to conversion to YLD rates using prevalence data for the full range of causes. Consequently, the high data requirements have become a significant obstacle for Chinese governments at all levels in measuring YLD, which in turn constrains their ability to measure HALE. The three-parameter YLD measurement model proposed in this study addresses the key issue of HALE measurement in China, particularly at the provincial and municipal levels. It is possible for provincial and municipal governments at all levels to obtain the IID from infectious disease surveillance data, PCDPF from chronic disease survey data, and U5MR from maternal and child surveillance data. These data can then be substituted into the ‘model (1)’ constructed in this study to obtain the YLD rate of the region by age. Once this has been done, the YLD rate can be combined with life expectancy to calculate the HALE of the region using the Sullivan method.

Currently, the GBD study leads in measuring HALE worldwide [9, 27, 28]. However, the measurement process of GBD’s HALE is quite complex, particularly the calculation process of YLD, which necessitates the use of global data resources and a sophisticated methodology. This makes it challenging to apply the methodology in different regions within China. The China YLD rate measurement model in this study was constructed on the basis of the GBD structure. Despite some deviations from GBD published values, this model greatly improves its applicability in China. This is due to the fact that the three predictor variables utilized in the model proposed in this study are more prevalent in China’s domestic provincial and municipal data resources, and the model is straightforward to implement. The IID data originates from China Infectious Disease Surveillance, and the U5MR data is derived from the Maternal and Child Surveillance and Coroner’s Surveillance programs, which collectively encompass all urban areas within the country. The PCDPF data is derived from two sources: the National Health Service Survey, conducted every five years in China, and the Chronic Disease and Risk Factor Surveillance Survey, conducted every three years [26].

All of the error values for YLD rates and HALEs are very small and approach zero, regardless of whether the models are sex specific or combined. This indicates a highly accurate model with estimates closely aligned with published GBD values. This indicates that the YLD measurement model constructed in this study is more reliable than several other YLD rate measures mentioned in the introduction. Nonetheless, we still present the four-parameter model for different genders in the Appendix. This is because the addition of the two-week incidence of impairment poisoning to the IID, PCDPF, and U5MR parameters led to a decrease in the total MAPE of the combined YLD rate and HALE by 14.12% and 32.18%, respectively.

The modeled errors by age showed that YLD MAEs increased and that MAPEs decreased with age, probably because YLD rates increase with age. Furthermore, the modeled errors by sex indicate that although the MAPEs of YLD rates and HALEs are lower for males than for females at almost all ages, the difference is almost negligible given the magnitude of YLD rates and HALEs values for both sexes. Similar findings were reported in previous studies performed by Jonker Marcel F. et al. [29] using the Bayesian random-effects approach to estimate healthy life expectancy.

The predictor variables we used to model the subsexed YLD rates in this study were as follows: only the PCDPF was split-sex. For IID and U5MR (including the two-week incidence of impairment poisoning for the four-parameter model in the Appendix), the combined rates were employed in the subsexed model. This is mainly because the MAE and MAPE of the sex-specific YLD rate are very close to those of the combined YLD rate in the sex-specific model, although only the PCDPF variable is sex specific. In fact, we also tried to include sex-specific IID and U5MR in the corresponding models, but the model errors hardly improved substantially. Another factor is that the aggregate values employed exhibit greater stability than the gender-specific values of the variables, without gender bias. In addition, using aggregate variables is more convenient than using gender-specific variables in model application. The official publicly released data for variables such as IID and the U5MR within China are all aggregated values, with few gender-specific values [30, 31].

Furthermore, it is worth noting that, compared with those of the GBD study, our study found narrower 95% UIs for the YLD rate and HALE model-fitted values. This disparity may be attributed to the differing calculation processes of UIs. The UIs of GBDs are based on a global data source and are estimated using many different estimation methods. There are large uncertainties inherent in this approach [32]. In contrast, our UIs are based on the YLD rates of GBD and their 95% UIs, computed using Monte Carlo methods. This is used to measure the overall reliability of the estimates.

This study employs the YLD rate data up to 2020, which does not incorporate the impact of the novel coronavirus (COVID-19) [33]. Notwithstanding this, the impact of the YLDs associated with the COVID-19 on the YLD rate in this study was minimal. This is due to the fact that the GBD 2021 results indicate that the majority of COVID-19-induced DALYs are deaths rather than YLDs [34]. Moreover, the global incidence of COVID-19-induced YLDs represents a mere 0.56% of the total YLD rate in 2020 and 1.6% in 2021. Indeed, it has been demonstrated that the impact of the COVID-19 pandemic on life expectancy in China has been relatively minimal [35].

The findings of this study indicate two potential avenues for future research. Firstly, it should be noted that the variables included in the model of this study are only applicable to the measurement of all-cause YLD rates in China. Nevertheless, the methodology employed in this study also indicates that other countries, particularly those with limited data resources, may utilize the same process to identify suitable variables for their regions and construct corresponding YLD rate measurement models. Secondly, the model proposed in this study is unable to differentiate between the YLD rates for different etiologies. Consequently, it is only capable of measuring the total YLD rate for all etiologies. In the future, further investigation can be conducted to ascertain the decomposition of the YLD measured by the model in this study into the YLD of the major etiologies.

Finally, the main limitation of this study is that our model simplifies the YLD rate measurement technique in GBD, relying on the GBD database. Hence, the accuracy of the estimates in this study is largely dependent on the reliability of YLD rate estimates in China provided by GBD. However, the GBD study is currently acknowledged as one of the most widely recognized and accepted research programs in HALE [36].

## Conclusion

Based on data from the GBD Study and domestic sources in China, this study constructed a three-input-parameter model to estimate YLD rates and HALEs by sex and age group in China. That is, to obtain the 21 age-specific YLD rates, the 3 predictor variables IID, PCDPF, and U5MR were inputted for some regions. These rates were combined with life tables to obtain HALEs. The results showed that the HALE measurement model we developed in China possesses a simple methodology, strong applicability and high accuracy. With limited data resources in China, our research provides a realistic and feasible solution for computing the national HALE, particularly at the provincial, city, and county levels.

### Electronic supplementary material

Below is the link to the electronic supplementary material.

Supplementary Material 1

## Data Availability

Domestic data available in China are from National Health Service Survey and Analysis Report , the China Health and Health Statistics Yearbook, and the China Statistical Yearbook . GBD 2019 data are from the Global Health Data Exchange query tool (https://vizhub.healthdata.org/gbd-results/).

## Abbreviations

HALE: Health-Adjusted Life Expectancy
YLD: Years lived with disability
GBD: Global Burden of Disease
MAE: Mean absolute error
MAPE: Mean absolute percentage error
WHO: World Health Organization
U5MR: Under-five mortality rate
IID: Incidence of infectious diseases
PCDPF: Incidence of chronic diseases among persons aged 15 and older
UIs: Uncertainty intervals
