# Supplementary material for: Construction and evaluation of a practical model for measuring health-adjusted life expectancy (HALE) in China
Source: BMC Public Health. 2024 Jun 22;24:1667. doi: 10.1186/s12889-024-19112-6 (PMC11193283; doi:10.1186/s12889-024-19112-6)
Supplement: Supplementary file 1 — Supplementary Material 1 [file 12889_2024_19112_MOESM1_ESM.pdf]

Supplementary appendix

Title: Construction and Evaluation of a Practical Model for Measuring Health-Adjusted Life Expectancy (HALE) in China

Journal: BMC Public Health

Author: San Qian Chen

Corresponding author: Song Bo Hu, Email: husbo0910@ncu.edu.cn. Phone: (+86)18379190848

Contents

Part I Methodological Steps of YLD Rate Model Construction and Evaluation

1. Collection and description of independent variables within China 3
   - 1.1 Collection of independent variables..........3
   - 1.2 Description of independent variables..........4
2. Treatment and screening of independent variables..........6
   - 2.1 Treatment of independent variables..........6
   - 2.2 Screening of independent variables..........7
3. Construction of the YLD rate model and parameter estimation..........9
   - 3.1 Simple modeling for measuring YLD rates by sex and age in China..........9
   - 3.2 Estimation of uncertainty intervals..........9
4. Assessment of model error..........11
   - 4.1 Sullivan method of measuring HALE..........11
   - 4.2 Calculation of indicators of error..........11

Part II Supplementation of model results

Table S4 Coefficients of the YLD rate measurement model by gender and age (4 independent variables)..........13

Table S6 Results of error assessment for HALE by gender and age group (4

independent variables) 16
Figure S2 Residuals of YLD rates by sex and age (4 independent variables) 17
Figure S3 Residuals for HALE by gender and age (4 independent variables) 17
Figure S4 Comparison of model-fitted values and true reference values of YLD rate for different age groups, 1990-2019 (3 Variables, Both) 18
Figure S5 Comparison of model-fitted values and true reference values of HALE for different age groups, 1990-2019 (3 Variables, Both)...19

Part I Methodological Steps of YLD Rate Model Construction and Evaluation

1. Collection and description of independent variables within China

1.1 Collection of independent variables

The Global Burden of Disease (GBD) 2019 divides all diseases into 3 major categories of 369 diseases, the three major categories are the first major category of diseases (communicable, maternal, neonatal, and nutritional diseases), the second major category of diseases (non-communicable diseases), and the third major category of diseases (injuries). diseases (injuries). Using Chinese data released in GBD 2019, we explored the relationship between the years lived with disability (YLD) rates and total prevalence of the three major categories of diseases across 22 age groups by gender based on multiple linear regression models. The R^2 results showed that, although individual age groups were more variable, the R^2 were very large overall, e.g., R^2 for the combined, male, and female all-ages were 0.98, 1.00, and 0.95, respectively. See Figure S1. This suggests that age-specific YLD rates are highly correlated with the overall prevalence of the three major disease categories.

Combining the actual available data resources within China at present, we selected three representative indicators - incidence rate of A and B statutory infectious diseases (IID), the prevalence of chronic diseases among persons aged 15 and older (PCDPF), and the two-week prevalence of impairment poisoning (TWPIP) - to represent the three major categories of diseases respectively. In addition, to improve the measurement accuracy of the model, we refer to the relevant covariates in the GBD technique as well as data accessibility, and screened 4 covariates, namely, under-5 mortality rate (U5MR), educational attainment per capita (EPC), urbanization rate (UR), and gross domestic product per capita (GDPPC), through Pearson correlation analysis. Table S1 shows the correlation coefficients of China's total YLD rates for different genders with the four covariates (U5MR, EPC, UR, GDPPC).

The seven independent variables were collected from the following sources:

PCDPF and TWPIP were derived from the National Health Service Survey and Analysis Report. IID and U5MR are from the China Health and Wellness Statistical Yearbook. EPC, UR and GDPPC are from the China Statistical Yearbook.

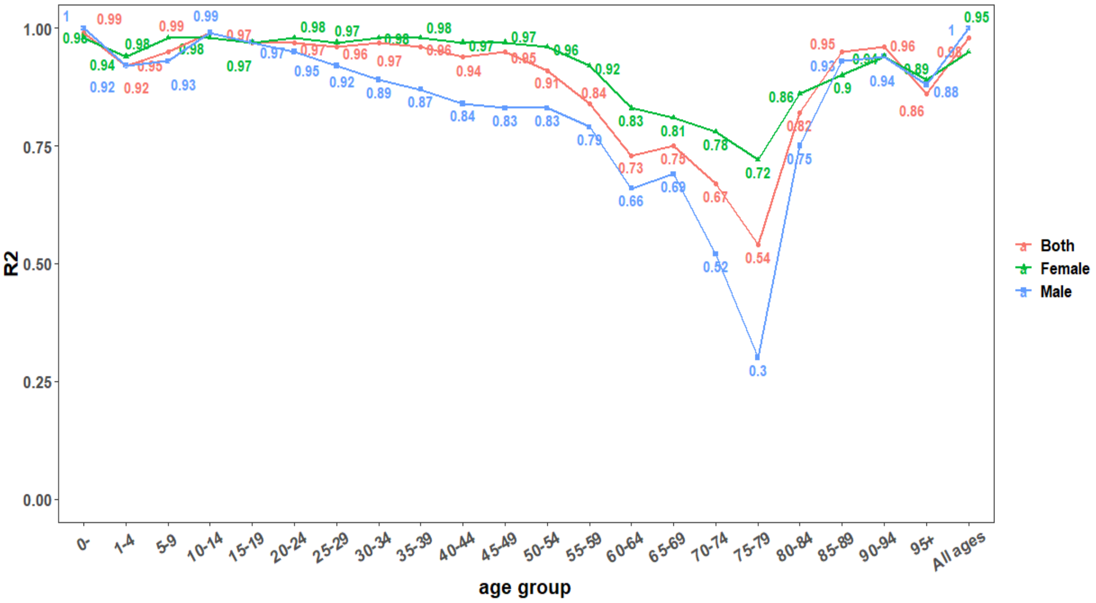

Figure S1　　R^2 of multiple linear regression models of age-specific YLD rates and total prevalence of three major disease categories in China

Table S1　　Correlation coefficients of total YLD rate with four covariates across gender in China

Covariates	Both YLD rate	Male YLD rate	Female YLD rate
EPC	0.9410	0.9412	0.9391
UR	0.9847	0.9835	0.9847
GDPPC	0.9840	0.9812	0.9865
U5MR	-0.9321	-0.9302	-0.9326

1.2 Description of independent variables

（1）PCDPF and TWPIP

Since 1993, China has conducted a large-scale nationwide health survey of its residents every five years and published that year's the National Health Service Survey and Analysis Report. Currently, the National Health Service Survey and Analysis

Report reports are available for six years (1993, 1998, 2003, 2008, 2013, 2018). In addition, the prevalence of chronic diseases in the National Health Service Survey Analysis Report is given for all ages in 1993, 1998, 2003, and 2008, while the last two years (2013, 2018) are given for the 15+ age group.

（2）IID

To effectively manage and control infectious diseases while safeguarding public health and safety, it is critical to promptly implement suitable preventive and control measures. In China, infectious diseases are classified into statutory infectious diseases, infectious diseases necessitating key monitoring, and other infectious diseases. Statutory infectious diseases are those that must be reported and controlled in accordance with procedures as stipulated by the Law of the People's Republic of China on Prevention and Control of Infectious Diseases. These diseases are highly contagious and harmful, posing a significant threat to public health security.

Statutory infectious diseases are categorized into three major groups, namely A, B and C, with 41 diseases. There are two statutory infectious diseases in category A, namely plague and cholera. There are 28 statutory infectious diseases in category B, namely, infectious atypical pneumonia, human infection with H7N9 avian influenza, AIDS, viral hepatitis, poliomyelitis, human infection with highly pathogenic avian influenza, measles, epidemic hemorrhagic fever, rabies, epidemic encephalitis B, dengue fever, anthrax, bacterial and amoebic dysentery, tuberculosis, typhoid fever and paratyphoid fever, epidemiological cerebral and spinal meningitis, whooping cough, diphtheria, neonatal tetanus, scarlet fever, brucellosis, gonorrhea, syphilis, leptospirosis, schistosomiasis, malaria, novel coronavirus infections, monkeypox. There are 11 statutory infectious diseases in category C, namely, influenza, mumps, rubella, acute hemorrhagic conjunctivitis, leprosy, epidemic and endemic typhus, black fever, encapsulated disease, filariasis, infectious diarrhoeal diseases other than cholera, bacterial and amoebic dysentery, typhoid fever and paratyphoid fever, and hand-foot-mouth disease.

The infectious diseases mentioned in this study are A and B statutory infectious diseases. The China Health Statistics Yearbook publishes the IID at the Chinese level

for the years 1950-2019 and annually publishes the IID for each of China's 31 provinces. In fact, the data on the IID are calculated from infectious disease surveillance data. Communicable disease surveillance covers Center for Disease Control and Prevention at the provincial, municipal and county levels throughout China.

（3）U5MR

China Health Statistics Yearbook released China's national-level U5MR from 1990-2019. In fact, the U5MR is calculated from maternal and child health surveillance data. China's maternal and child health monitoring covers 140 million people in 31 provinces.

（4）EPC, UR and GDPPC

The China Statistical Yearbook publishes GDPPC from 1978 to 2022 and the UR from 1949 to 2022. In addition, the China Statistical Yearbook published the population by gender and educational attainment from 1987-2022, and then according to the formula “EPC = [number of people not attending school * 0 + number of elementary school * 6 + number of middle schools * 9 + number of senior high schools * 12 + (college + bachelor's degree + postgraduate students) * 16] / number of people aged 6 years and above ”, to calculate the EPC in 1987-2022.

2. Treatment and screening of independent variables

2.1 Treatment of independent variables

（1）Spline interpolation

Spline interpolation was used to fill in the missing years. The PCDPF was interpolated using cubic spline interpolation, based on data from six years (1993, 1998, 2003, 2008, 2013, and 2018), and smoothed data were generated for the years 1990-2019 by using the cubicspline function in the pracma package of the R language. The PCDPF, by sex, is filled in using the same methodology. The TWPIP was interpolated using quadratic spline interpolation, i.e., based on these six years of data using the spline function in the R language splines package to generate smoothed data for the years 1990-2019.

（2）Age group conversions

In order to standardize the criteria, we converted the chronic disease prevalence rate of the all-age population into the chronic disease prevalence rate of the population aged 15 years and above in 1993, 1998, 2003, and 2008, based on the age group composition ratio of the surveyed population and the chronic disease prevalence rate of the age group.

2.2 Screening of independent variables

We fitted a multiple linear regression model of the seven predictor variables collected and screened above (IID, PCDPF, TWPIP, U5MR, EPC, UR, GDPPC) to the age-specific YLD rates published by GBD. Keeping the three variables of the IID, PCDPF, TWPIP fixed, and adding four covariates (U5MR, EPC, UR, GDPPC), there are a total of 16 combinations, as shown in Table S2.

We performed multicollinearity diagnostics on these 16 models and excluded models with variance expansion factors greater than 10 in the independent variables, leaving four models, namely, Model 12, Model 13, Model 14, and Model 16. With the guidelines of the smaller error of YLD rate and HALE, the smaller number of independent variables, and the strongest accessibility of the independent variables at the provincial, city, and county level (see later for the calculation process of YLD rate and HALE), model 12 was finally selected. Four independent variables were screened out (IID, PCDPF, TWPIP, U5MR). See Table S3. Under the premise of ensuring the accuracy of the model, to further enhance the applicability of the model, this paper shows the 3-parameter model (IID, PCDPF, and U5MR) in the main text, and shows the results of a 4-parameter model (IID, PCDPF, TWPIP and U5MR) results. The entire filtering process uses the R language, and the specific code is available at https://github.com/sanqian-chen/Filtering-Variables。

The maximum, minimum, median, and mean of the two-week prevalence of impairment poisoning were 0.0059, 0.0038, 0.0047, and 0.0048, respectively. The maximum, minimum, median, and mean for the remaining three independent variables (PCDPF, IID, and U5MR) are shown in Table 1 in the main text.

Table S2 Variance Expansion Factors for the Dependent Variables of the 16 Combined Models

Model	X1	X2	X3	X4	X5	X6	X7
Model 1	2.59	103.16	6.13	42.84	15.40	163.79	418.67
Model 2	1.70	10.47	3.02	42.72	13.42	66.80	
Model 3	2.07	67.14	4.14	27.25	14.96		170.76
Model 4	2.58	95.08	5.48	40.49		159.14	364.75
Model 5	2.58	101.14	6.13		14.56	104.19	417.47
Model 6	1.70	5.62	3.01	9.55	12.98		
Model 7	1.62	10.04	2.98	40.44		64.63	
Model 8	2.06	63.69	3.80	26.48			148.12
Model 9	1.68	6.71	3.02		12.70	14.94	
Model 10	1.62	27.22	3.11		14.54		59.84
Model 11	2.57	90.47	5.46			104.07	364.34
Model 12	1.61	4.47	2.97	2.84			
Model 13	1.61	5.62	2.94		3.85		
Model 14	1.61	6.67	2.97			4.53	
Model 15	1.62	18.41	2.53				15.87
Model 16	1.60	2.31	1.72				

X1, X2, X3, X4, X5, X6 and X7 represent, respectively, the IID, PCDPF, TWPIP, U5MR, EPC, UR and GDPPC

Table S3 Total MAE and MAPE for YLD rates and HALE for the 4 combination models

Model	YLD		HALE	
	MAE	MAPE	MAE	MAPE
Model 12	0.0005	0.511	0.0144	0.0723
Model 13	0.0007	0.796	0.021	0.0893
Model 14	0.0005	0.425	0.0141	0.0731
Model 16	0.0012	1.580	0.0383	0.1330

3. Construction of the YLD rate model and parameter estimation

3.1 Simple modeling for measuring YLD rates by sex and age in China

In this study, the YLD rate models for 4-parameter and 3-parameter are given, and the 3-parameter YLD rate model is shown in the main text, followed by the 4-parameter model.

The model is based on the input of four indicators, namely, IID, PCDPF, U5MR and TWPIP, in order to obtain the YLD rates for the 21 age groups, as follows:

（1）Twenty-one multiple linear regression models were constructed with the four rate indicators as independent variables and the YLD rates of each of the 21 age groups as dependent variables:

$$\Gamma(Y_i) = \alpha_i + d_i X_1 + e_i X_2 + f_i X_3 + g_i X_4 \qquad (5)$$

Where, Y_i is the YLD rate for 21 age groups (0 year old group,1-4 year old group,5-9 year old group,10-14 year old group,......,90-94 year old group,95+ year old group), X_1、X_2、X_3、X_4 are the IID, PCDPF, U5MR and TWPIP respectively. α_i are the intercepts, d_i、e_i、f_i、g_i are the regression coefficients of the corresponding independent variables, $\Gamma(Y_i)$ and are Logit transformations.

In addition, this study also compared three ways of using raw values, Ln transformation, and Logit transformation for Y_i, but Logit transformation was finally chosen. The reasons are as follows: firstly, Logit transform has small error; secondly, the model may have negative numbers when extrapolating in prediction, and the use of Logit transform can circumvent the existence of negative numbers.

（2）In constructing the gender-specific model (5), except for the PCDPF, the other three independent variables were modeled by directly replacing the gender-specific rate with the both rate.

The model was constructed using R. The code is available at :https://github.com/sanqian-chen/Filtering-Variables。

3.2 Estimation of uncertainty intervals

We assessed the uncertainty of the Model coefficients, YLD rate and HALE

estimates based on the uncertainty of the input data from GBD. Generation of 95% uncertainty intervals (UI) based on multivariate normal distribution using Monte Carlo method. Uncertainty was obtained by sampling from a multivariate normal distribution constructed from GBD YLD rates and their 95% UI.

We assumed GBD data are normally distributed with mean values equal to the point estimates. We assume that the GBD YLD rates for each year, after Logit transformation, obey a multivariate normal distribution with 21 dimensions. For each year and sex we proceeded as follows:

1）The YLD rate and its 95% UI for different genders and 21 age groups from 1990-2019 in the GBD database were logit transformed to construct a multivariate normal distribution. The YLD rate and 95% UI stated in this subsection are the values after Logit transformation.

2）We construct a multivariate normal distribution for each of the years 1990-2019, with each of the 21 age groups of each multivariate normal distribution simultaneously randomized to 1000 $\Gamma(\mathrm{Y}_i)$. The multivariate normal distribution requires two parameters, a vector of means and a 21-dimensional covariance matrix.

3）The mean vector is the YLD rate for each of the 21 age groups for each year.

4）The construction of the 21-dimensional covariance matrix is divided into 3 parts:

① Each age group has its own covariance, or variance. We inverted two standard deviations based on the GDB YLD rate and its 95% uncertainty interval based on the "$\mu \pm 1.96 \times \sigma$"formula. The mean back-squared of the two standard deviations was calculated as each age group's own covariance for each year.

② Covariance and correlation coefficient for two-by-two age groups. We constructed 21 samples with a sample size of 30 in 21 age categories YLD rate from 1990-2019, with each of the 21 samples corresponding to 21 age groups, and then calculated the covariance and correlation coefficients for the two-by-two age groups.

③ Construct the 21-dimensional covariance matrix for each year. We assumed that the covariance and correlation coefficients for the two-by-two age groups remained constant, and changed the variance of the main diagonal to produce a covariance matrix for the 21 dimensions for each year.

5）The obtained 30 mean vectors and covariance matrices were constructed into 30 multivariate normal distributions, each with 21 age groups.1000 $\Gamma(Y_i)s$ are randomly selected simultaneously from each age group of each multivariate normal distribution. That is, we now have a total of 30 years of data, each with 21 age groups, and each with another 1000 $\Gamma(Y_i)s$.

6）Model (1) was fitted to the data obtained with the three independent variables to obtain 1000 copies of model (1), and the 95% UI of the model coefficients β_i、a_i、b_i and c_i were obtained by calculating the 2.5th and 97.5th quartiles.

7）1000 copies of model (1) yields 1000 copies of the YLD rate model fit, which in combination with the GBD's life table yields 1000 copies of the HALE model fit, taking the 2.5th quartile and the 97.5th quartile, and thus calculating the 95% UI.

8）Use the mvrnorm function in the MASS package of the R language to realize 1000 random samples from a multivariate normal distribution. The specific code is available at :https://github.com/sanqian-chen/Filtering-Variables/tree/Construction-model。

4. Assessment of model error

4.1 Sullivan method of measuring HALE

Like life expectancy, HALE is not affected by the age structure of the population. Its calculation formula:

$$HALE_i = \frac{1}{l_i}[\sum_{i=0}^{w} L_i \times (1 - Y_i)] \qquad (7)$$

Where, l_i denotes the number of survivors in age group i, w denotes the largest age group (the 95+ year group), L_i denotes the number of survivor years in age group i, and Y_i is the age-specific YLD rate measured by model (6). l_i and L_i and are both derived from the life table data in GBD 2019.

4.2 Calculation of indicators of error

In this study, we evaluate the accuracy of this simple model in terms of two

dimensions: the YLD rate and the HALE. The YLD rate and HALE published by GDB are used as the real reference values to estimate the error between the calculated values of our model and the real reference values published by GBD. The evaluation methodology is consistent with the main text and will not be repeated here.

Part II Supplementation of model results

Table S5 Results of error assessment of YLD rates by gender and age (4 independent variables)

Age group	Male			Female			Both		
	MAE	MAPE	RMSE	MAE	MAPE	RMSE	MAE	MAPE	RMSE
0-	0.0004	1.5230	0.0005	0.0004	1.4797	0.0005	0.0004	1.4965	0.0005
1-4	0.0003	0.9858	0.0003	0.0003	1.3466	0.0004	0.0003	1.1334	0.0004
5-9	0.0002	0.7187	0.0003	0.0002	0.8461	0.0003	0.0002	0.7686	0.0003
10-14	0.0001	0.2664	0.0001	0.0001	0.3088	0.0002	0.0001	0.2959	0.0001
15-19	0.0002	0.3399	0.0002	0.0003	0.4463	0.0003	0.0002	0.4012	0.0003
20-24	0.0004	0.7896	0.0005	0.0006	0.8712	0.0008	0.0005	0.8063	0.0006
25-29	0.0005	0.723	0.0006	0.0008	0.9136	0.0009	0.0006	0.7933	0.0007
30-34	0.0004	0.6287	0.0005	0.0009	0.9024	0.0010	0.0006	0.7647	0.0007
35-39	0.0004	0.5664	0.0005	0.0009	0.8373	0.0010	0.0006	0.6914	0.0007
40-44	0.0005	0.5204	0.0006	0.0008	0.7159	0.0010	0.0006	0.6281	0.0007
45-49	0.0004	0.4284	0.0005	0.0007	0.5335	0.0008	0.0005	0.4284	0.0006
50-54	0.0004	0.3572	0.0005	0.0007	0.4513	0.0008	0.0005	0.4103	0.0006
55-59	0.0004	0.2865	0.0004	0.0006	0.3488	0.0007	0.0004	0.2909	0.0005
60-64	0.0003	0.1720	0.0003	0.0004	0.2117	0.0005	0.0003	0.1847	0.0004
65-69	0.0003	0.1640	0.0004	0.0004	0.1633	0.0004	0.0003	0.1455	0.0004
70-74	0.0005	0.2225	0.0006	0.0005	0.2054	0.0006	0.0005	0.1990	0.0005
75-79	0.0007	0.2752	0.0008	0.0007	0.2308	0.0008	0.0006	0.2164	0.0007
80-84	0.0007	0.2637	0.0009	0.0008	0.2429	0.0009	0.0007	0.2387	0.0008
85-89	0.0008	0.2562	0.0010	0.0009	0.2436	0.0011	0.0008	0.2397	0.0010
90-94	0.0011	0.3303	0.0013	0.0010	0.2736	0.0013	0.0010	0.2669	0.0012
95+	0.0013	0.3729	0.0016	0.0014	0.3322	0.0017	0.0013	0.3283	0.0017
Total	0.0005	0.4853	0.0006	0.0006	0.5669	0.0008	0.0005	0.5109	0.0006

mean absolute error (MAE), root mean square error (RMSE), mean absolute percentage error (MAPE)，MAE and RMSE are in units of 1 and MAPE is in %.

Table S4 Coefficients of the YLD rate measurement model by gender and age (4 independent variables)

Age groups	Male					Female					Both				
	α_i	d_i	e_i	f_i	g_i	α_i	d_i	e_i	f_i	g_i	α_i	d_i	e_i	f_i	g_i
0-	1.9242(1.4112,2.4437)	20.2123(-103.6061,135.5415)	-0.0017(-0.6393,0.7229)	-3.7102(-6.9046,-0.3125)	-12.9567(-93.5749,67.5938)	1.9634(1.4107,2.4954)	8.1690(-104.2254,123.4181)	-0.0834(-0.7432,0.6447)	-4.2446(-7.4508,-0.8085)	-7.4260(-83.6103,72.9163)	1.9403(1.4353,2.4426)	14.3532(-112.2463,122.6064)	-0.0360(-0.6978,0.6727)	-3.9488(-6.9077,-0.4077)	-10.2469(-89.6014,70.1843)
1-4	1.9421(1.4303,2.4402)	-10.0435(-133.4594,107.5222)	-0.1802(-0.8284,0.5191)	-2.6328(-5.6826,0.5823)	5.6542(-74.9293,83.2563)	2.0444(1.5054,2.5548)	-15.7958(-135.9043,104.7909)	-0.2613(-0.9069,0.4538)	-3.3724(-6.4635,-0.0532)	6.5530(-72.3011,88.3903)	1.9846(1.4524,2.4728)	-12.3052(-143.39,100.5432)	-0.2118(-0.8402,0.4622)	-2.9370(-5.9526,0.3832)	6.1138(-74.5928,89.1634)
5-9	1.8240(1.2995,2.3269)	-4.0115(-124.5834,115.0798)	-0.1335(-0.7835,0.589)	-1.8628(-4.8986,1.4372)	3.5761(-77.0736,80.4822)	1.8937(1.3856,2.4046)	-2.4483(-120.0469,113.7549)	-0.1725(-0.8002,0.5561)	-2.2842(-5.3085,0.8592)	2.5903(-75.3493,82.2696)	1.8530(1.3277,2.3456)	-3.0079(-134.3433,108.1133)	-0.1484(-0.7867,0.5313)	-2.0254(-5.0168,1.3377)	3.1794(-76.9227,84.7083)
10-14	1.6361(1.1422,2.1348)	2.7550(-116.6062,115.3859)	-0.0593(-0.692,0.6503)	-0.8351(-3.9106,2.4619)	-0.9385(-78.8908,75.8508)	1.6043(1.093,2.1198)	4.4258(-111.6995,122.2066)	-0.0811(-0.7227,0.6445)	-0.8610(-3.8571,2.3225)	-3.1945(-83.9238,77.1383)	1.6202(1.1158,2.0882)	3.8443(-126.2406,112.815)	-0.0680(-0.7015,0.6251)	-0.8668(-3.7462,2.356)	-2.0649(-80.8784,81.1841)
15-19	1.5401(1.0509,2.0438)	6.3101(-113.0615,117.4732)	-0.0506(-0.6614,0.6105)	-0.7167(-3.7119,2.6382)	-2.8894(-79.0722,73.8232)	1.4843(0.942,2.0014)	3.4105(-111.815,120.4928)	-0.0991(-0.7711,0.6495)	-1.4053(-4.5142,1.9176)	-5.3507(-85.0564,77.6356)	1.5111(1.0043,1.9742)	4.7866(-121.3507,109.9299)	-0.0686(-0.7166,0.6241)	-1.0908(-4.0064,2.2095)	-4.2838(-81.8554,79.8817)
20-24	1.4729(1.0176,1.9346)	7.7523(-103.4078,109.688)	-0.0610(-0.6557,0.5894)	-1.3030(-4.0865,1.8622)	-3.9953(-75.5839,67.8309)	1.4032(0.8865,1.8907)	2.3732(-104.827,113.8616)	-0.1027(-0.7529,0.6067)	-2.1539(-5.2161,1.0248)	-5.5893(-80.3783,73.4146)	1.4336(0.9729,1.8905)	4.7046(-114.9944,105.7469)	-0.0725(-0.6919,0.5795)	-1.7095(-4.3964,1.3543)	-4.9034(-79.575,75.2918)

25-29	1.4069(0.9578,1.8624)	0.8797(-111.2775,104.2213)	-0.0816(-0.6641,0.5812)	-1.3027(-4.1021,1.6693)	-1.8060(-75.3417,69.9856)	1.3065(0.7804,1.8231)	1.9630(-108.5803,116.6695)	-0.0833(-0.7592,0.6251)	-2.0362(-5.1993,1.2568)	-6.1027(-82.1196,73.5686)	1.3566(0.8914,1.8261)	1.0249(-120.6113,101.9824)	-0.0813(-0.7008,0.5921)	-1.6537(-4.4602,1.4554)	-4.5387(-82.0875,74.8102)
30-34	1.3486(0.8641,1.8476)	-4.9617(-118.2742,107.1775)	-0.0916(-0.706,0.5819)	-1.1990(-4.0836,1.8132)	1.3220(-74.7633,73.7847)	1.2261(0.6932,1.7645)	1.1487(-111.0365,115.2938)	-0.0654(-0.7493,0.6503)	-1.8998(-5.0996,1.44)	-5.2118(-83.3443,75.2729)	1.2841(0.7909,1.7657)	-1.2814(-130.2832,105.546)	-0.0767(-0.7136,0.617)	-1.5384(-4.4585,1.6473)	-2.3191(-81.1384,79.559)
35-39	1.2957(0.8316,1.7716)	-5.9203(-115.0789,105.7627)	-0.1021(-0.7005,0.5381)	-1.1779(-3.9893,1.71)	1.2405(-73.852,69.7102)	1.1775(0.6542,1.7019)	-0.6466(-110.2094,110.6069)	-0.0748(-0.739,0.6241)	-1.8446(-4.9871,1.4165)	-5.7972(-82.6009,73.1376)	1.2378(0.7542,1.7104)	-2.5144(-124.1614,103.007)	-0.0905(-0.7206,0.5816)	-1.5239(-4.3775,1.5905)	-3.1943(-80.4879,78.6658)
40-44	1.2154(0.7481,1.7002)	-5.3675(-117.8588,105.2433)	-0.0956(-0.7145,0.5264)	-0.9672(-3.8691,1.9892)	1.6649(-75.6519,76.4045)	1.1049(0.5696,1.6436)	-1.4296(-112.8905,111.8565)	-0.0663(-0.7386,0.6339)	-1.5435(-4.6944,1.7593)	-5.4166(-83.9004,74.8427)	1.1576(0.6763,1.6265)	-3.0263(-126.5429,106.5181)	-0.0788(-0.7071,0.5887)	-1.2375(-4.1181,1.9306)	-2.1313(-79.394,79.2476)
45-49	1.1448(0.6742,1.6383)	-4.7712(-120.5133,109.596)	-0.0828(-0.7174,0.5654)	-0.8192(-3.8065,2.1935)	0.4602(-78.0577,77.4664)	1.0329(0.5064,1.5827)	-0.2049(-111.9202,114.3517)	-0.0625(-0.7435,0.6513)	-1.2641(-4.4491,2.0353)	-5.3506(-83.6305,74.3712)	1.0840(0.588,1.5549)	-1.7298(-127.4325,110.3503)	-0.0697(-0.7139,0.6076)	-0.9993(-3.9373,2.2866)	-2.4896(-81.4086,81.5549)
50-54	1.0672(0.5739,1.5805)	-4.5186(-122.0451,116.9338)	-0.0668(-0.721,0.6275)	-0.6670(-3.6776,2.4282)	-0.6661(-81.8736,77.4292)	0.9551(0.4324,1.4999)	-0.3575(-110.6961,112.2472)	-0.0541(-0.7315,0.6472)	-1.0741(-4.2572,2.2356)	-5.4799(-83.2824,74.2157)	1.0097(0.5376,1.4932)	-1.6087(-129.0447,107.0687)	-0.0623(-0.7042,0.6288)	-0.8227(-3.8712,2.4388)	-3.6059(-84.552,81.4633)
55-59	0.9822(0.4873,1.5045)	-4.5814(-122.7129,115.0124)	-0.0535(-0.6938,0.6454)	-0.5609(-3.6546,2.6312)	-1.8206(-81.0859,74.7751)	0.8845(0.3536,1.4641)	-2.2375(-114.6095,111.7761)	-0.0471(-0.7328,0.6829)	-0.8314(-3.9757,2.5199)	-5.6175(-84.9965,75.7951)	0.9336(0.4523,1.4179)	-3.4380(-128.3292,107.549)	-0.0522(-0.6901,0.6635)	-0.6573(-3.7714,2.5504)	-4.0241(-84.5107,78.6885)
60-64	0.8865(0.4077,1.4107)	-4.5967(-121.0599,117.3246)	-0.0324(-0.6906,0.6243)	-0.3613(-3.5011,2.8507)	-3.0799(-85.8059,71.1144)	0.8034(0.2938,1.395)	-2.2546(-115.6541,110.3892)	-0.0372(-0.7466,0.6627)	-0.5451(-3.7304,2.8036)	-6.7346(-85.9518,72.0468)	0.8441(0.35,1.3358)	-4.1307(-129.1721,110.6637)	-0.0367(-0.6822,0.6825)	-0.4342(-3.5426,2.9135)	-4.5273(-83.4921,76.3264)

65-69	0.7769(0.2962,1.2792)	-2.2479(-116.9208,109.6163)	-0.0030(-0.6595,0.701)	-0.2297(-3.4018,3.0957)	-5.5521(-83.8968,70.9684)	0.7085(0.2291,1.2581)	0.3556(-108.7862,113.9983)	-0.0328(-0.7375,0.5886)	-0.4033(-3.602,2.7209)	-8.8675(-87.3691,65.4275)	0.7447(0.2317,1.2256)	-1.2913(-114.3104,111.5659)	-0.0245(-0.6509,0.6663)	-0.3538(-3.3922,2.7517)	-7.1530(-83.0842,70.7111)
70-74	0.6767(0.1831,1.1699)	0.6918(-124.2442,113.0041)	0.0025(-0.6703,0.7177)	-0.1148(-3.3588,3.2277)	-8.1397(-83.9823,73.1217)	0.6111(0.1462,1.1511)	2.1753(-106.7551,117.8985)	-0.0352(-0.7292,0.5741)	-0.2752(-3.4276,2.7139)	-9.6572(-87.7436,61.2354)	0.6457(0.1301,1.1381)	1.0635(-110.6812,114.8833)	-0.0220(-0.6559,0.6637)	-0.2682(-3.3967,2.9244)	-8.8656(-87.3964,69.9176)
75-79	0.5861(0.0318,1.1199)	1.2220(-124.4997,126.4781)	-0.0085(-0.7164,0.7767)	0.0794(-3.5251,3.623)	-7.5756(-89.5503,75.9959)	0.5157(0.0576,1.0215)	2.1569(-112.6968,127.3912)	-0.0421(-0.7222,0.5992)	-0.0986(-3.3679,2.972)	-8.4623(-85.6832,62.7451)	0.5493(0.0477,1.0628)	1.6943(-118.248,121.5057)	-0.0270(-0.6874,0.6622)	-0.1057(-3.1948,3.1045)	-8.0178(-90.0908,72.2367)
80-84	0.4917(-0.0579,1.0445)	2.0857(-122.8495,130.5279)	-0.0255(-0.7736,0.7421)	0.3110(-3.243,3.6604)	-6.2858(-93.5547,81.7281)	0.4031(-0.1231,0.98)	2.3784(-109.5871,125.0037)	-0.0459(-0.7877,0.7002)	0.1160(-3.4442,3.5933)	-5.6533(-91.7876,75.5422)	0.4405(-0.0974,0.9864)	2.8158(-122.3236,125.2109)	-0.0342(-0.7674,0.6835)	0.1122(-3.2772,3.5905)	-6.2929(-91.123,81.0322)
85-89	0.4116(-0.1621,0.9658)	2.3088(-131.1292,138.7113)	-0.0351(-0.7895,0.7146)	0.4122(-3.351,4.01)	-3.3846(-90.516,82.3455)	0.3275(-0.2278,0.8701)	4.9863(-123.7331,127.0267)	-0.0685(-0.7774,0.6533)	-0.0156(-3.468,3.3945)	-3.9605(-87.8164,80.484)	0.3531(-0.2228,0.8898)	3.2827(-129.5894,138.4086)	-0.0498(-0.774,0.7321)	0.1011(-3.5456,3.6504)	-3.5589(-91.0903,86.7548)
90-94	0.3503(-0.2095,0.9068)	5.9974(-127.4353,139.6026)	-0.0507(-0.8005,0.7049)	0.5471(-3.1581,4.111)	-2.8513(-91.973,85.7433)	0.2719(-0.2407,0.7986)	9.9632(-117.7907,129.9633)	-0.1061(-0.801,0.5869)	-0.2049(-3.6162,3.2246)	-3.7498(-83.5087,79.9396)	0.2753(-117.7907,129.9633)	6.0678(-118.8221,139.3178)	-0.0906(-0.8632,0.6516)	0.0197(-3.5954,3.5583)	-1.0162(-84.9014,87.7201)
95+	0.2942(-0.2787,0.8718)	14.3392(-123.7714,150.8877)	-0.0851(-0.8386,0.6891)	0.5227(-3.1125,4.2623)	-6.4560(-98.4813,81.9535)	0.2134(-0.3154,0.7394)	17.8203(-101.8107,154.3459)	-0.1431(-0.8731,0.5391)	-0.5023(-4.1398,2.8572)	-5.8265(-89.0366,75.6081)	0.2244(-0.3459,0.7689)	17.8558(-112.6747,144.0434)	-0.1488(-0.8801,0.6134)	-0.3659(-3.7422,3.3441)	-6.1641(-90.7742,82.4816)

Values in parentheses are 95% uncertainty intervals.

Table S6 Results of error assessment for HALE by gender and age group (4 independent variables)

Age group	Male			Female			Both		
	MAE	MAPE	RMSE	MAE	MAPE	RMSE	MAE	MAPE	RMSE
0-	0.0182	0.0288	0.0211	0.0384	0.0581	0.046	0.0273	0.0423	0.0324
1-4	0.0183	0.0287	0.0214	0.0388	0.0584	0.0465	0.0276	0.0424	0.0327
5-9	0.0176	0.0292	0.0206	0.0379	0.0602	0.0455	0.0267	0.0434	0.0318
10-14	0.0168	0.0301	0.0196	0.037	0.0636	0.0445	0.0258	0.0453	0.0308
15-19	0.0165	0.0323	0.0193	0.0366	0.0685	0.044	0.0256	0.0490	0.0305
20-24	0.016	0.0344	0.0186	0.0356	0.0728	0.0428	0.0247	0.0518	0.0294
25-29	0.0142	0.0338	0.0166	0.0326	0.0734	0.0394	0.0225	0.0522	0.0269
30-34	0.0122	0.0324	0.0145	0.0291	0.0727	0.0354	0.0195	0.0502	0.0234
35-39	0.0103	0.0309	0.0123	0.025	0.0699	0.0306	0.0167	0.0484	0.0204
40-44	0.0084	0.0287	0.0104	0.0208	0.066	0.0258	0.0137	0.0452	0.0171
45-49	0.0067	0.0265	0.0085	0.0169	0.0619	0.0215	0.0111	0.0422	0.0142
50-54	0.006	0.0284	0.0076	0.014	0.0598	0.0179	0.0093	0.0420	0.012
55-59	0.006	0.0344	0.0069	0.0115	0.059	0.0148	0.0079	0.0426	0.010
60-64	0.0065	0.0471	0.0075	0.0099	0.0625	0.0125	0.0076	0.0512	0.0091
65-69	0.0069	0.0642	0.008	0.0095	0.0758	0.0116	0.0074	0.0643	0.0088
70-74	0.0064	0.0813	0.0078	0.0087	0.0923	0.0106	0.0069	0.0799	0.0081
75-79	0.0056	0.0971	0.0067	0.0075	0.108	0.0093	0.0061	0.0963	0.0071
80-84	0.0043	0.1082	0.0051	0.0061	0.1241	0.0076	0.0051	0.1141	0.006
85-89	0.0031	0.1177	0.0037	0.0047	0.1386	0.0063	0.0041	0.1324	0.005
90-94	0.0034	0.1622	0.004	0.0043	0.1757	0.0054	0.0037	0.1588	0.0047
95+	0.0031	0.2108	0.0037	0.0038	0.2389	0.0047	0.0036	0.2327	0.0045
Total	0.0098	0.0613	0.0116	0.0204	0.0886	0.0249	0.0144	0.0727	0.0174

mean absolute error (MAE), root mean square error (RMSE), mean absolute percentage error (MAPE), MAE and RMSE in years and MAPE in %.

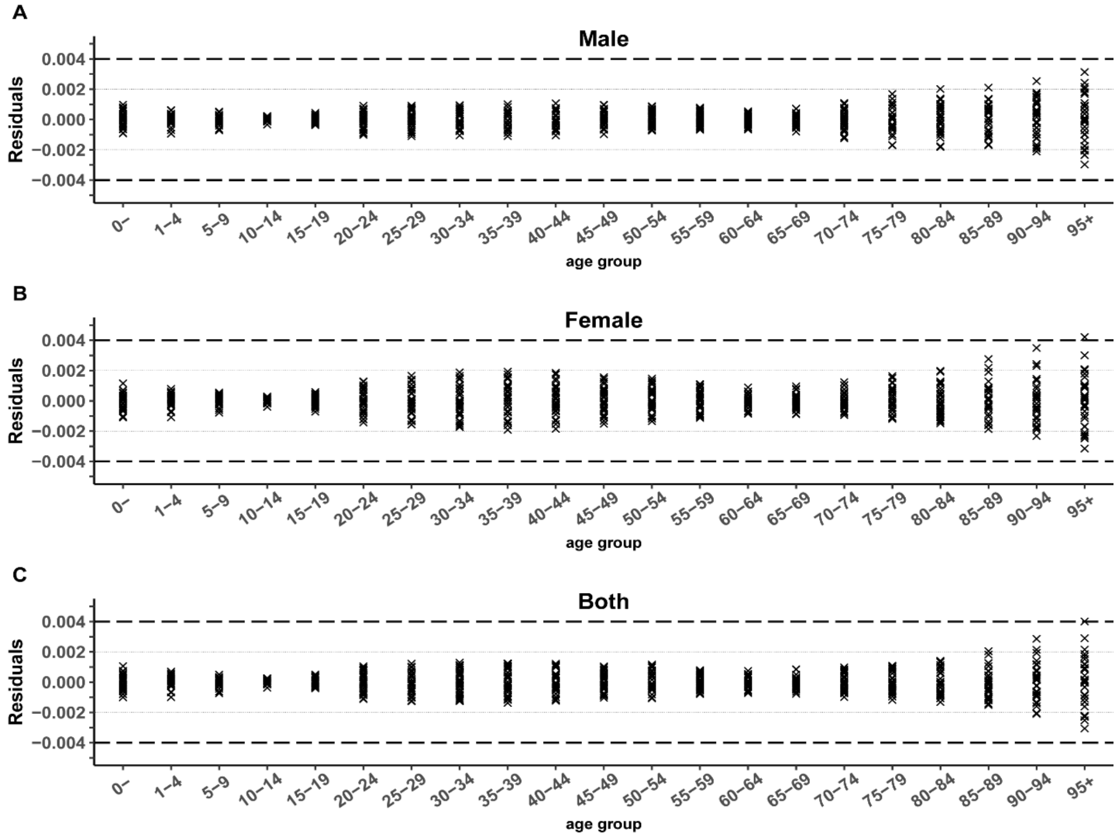

Figure S2 Residuals of YLD rates by sex and age (4 independent variables)

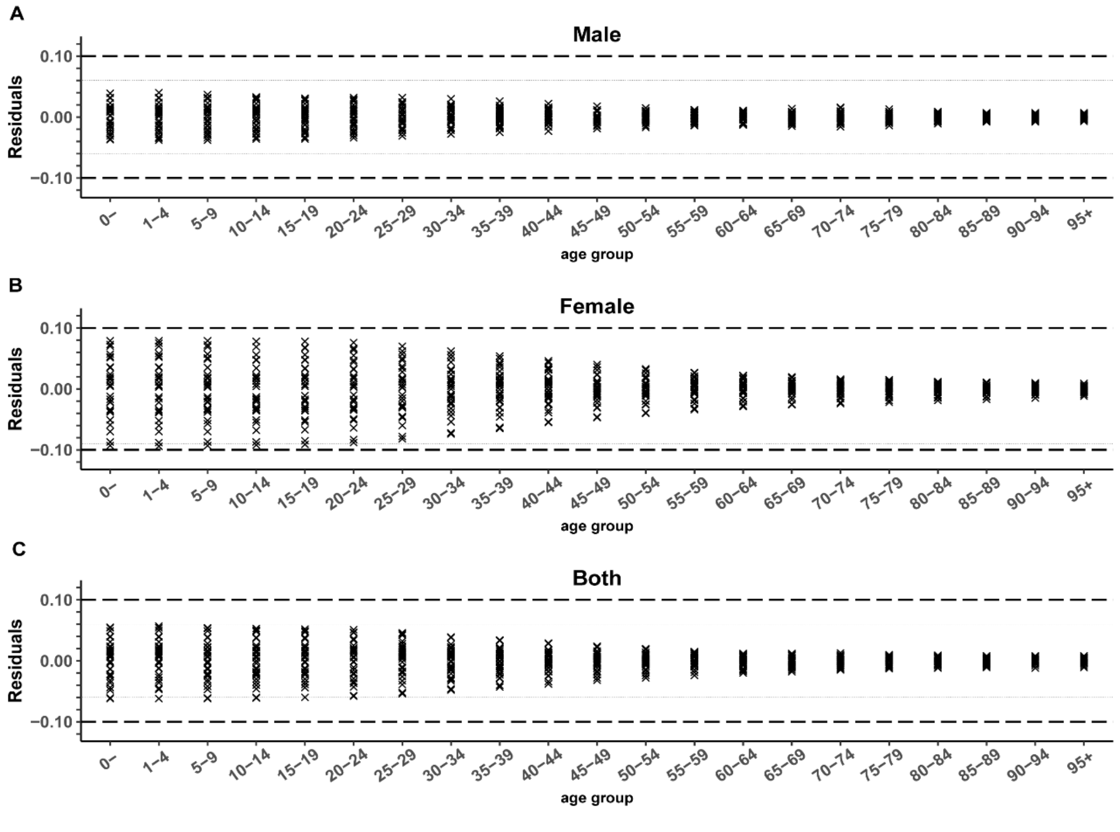

Figure S3 Residuals for HALE by gender and age (4 independent variables)

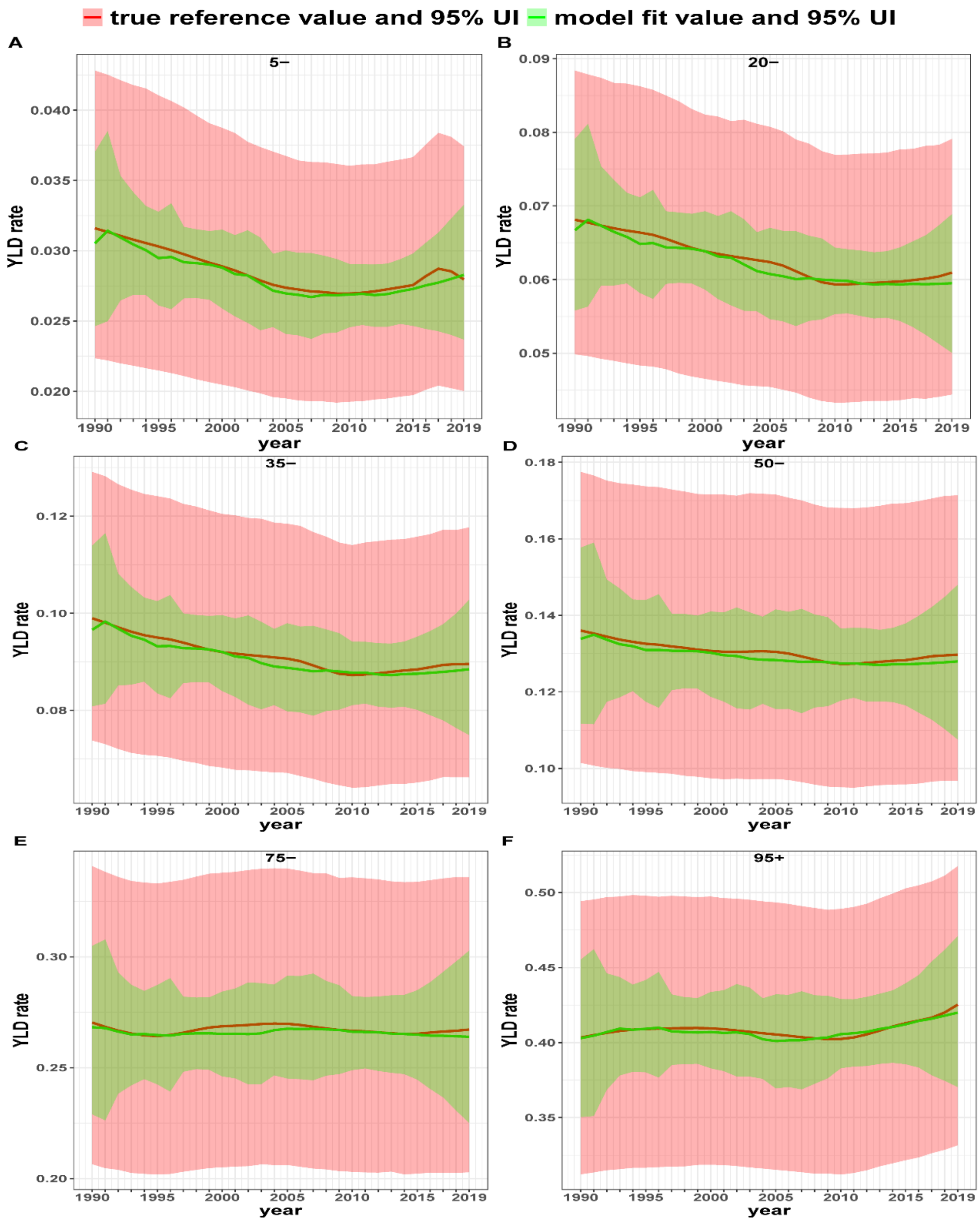

Figure S4 Comparison of model-fitted values and true reference values of YLD rate for different age groups, 1990-2019 (3 Variables, Both)

(A), (B), (C), (D), (E), (F) indicate comparisons of model-fitted values and true reference values of the combined YLD rate for the 5-9 age group, 20-24 age group, 35-39 age group, 50-54 age group, 75-79 age group and 95+ age group separately. Shaded areas indicate the corresponding 95% uncertainty intervals.

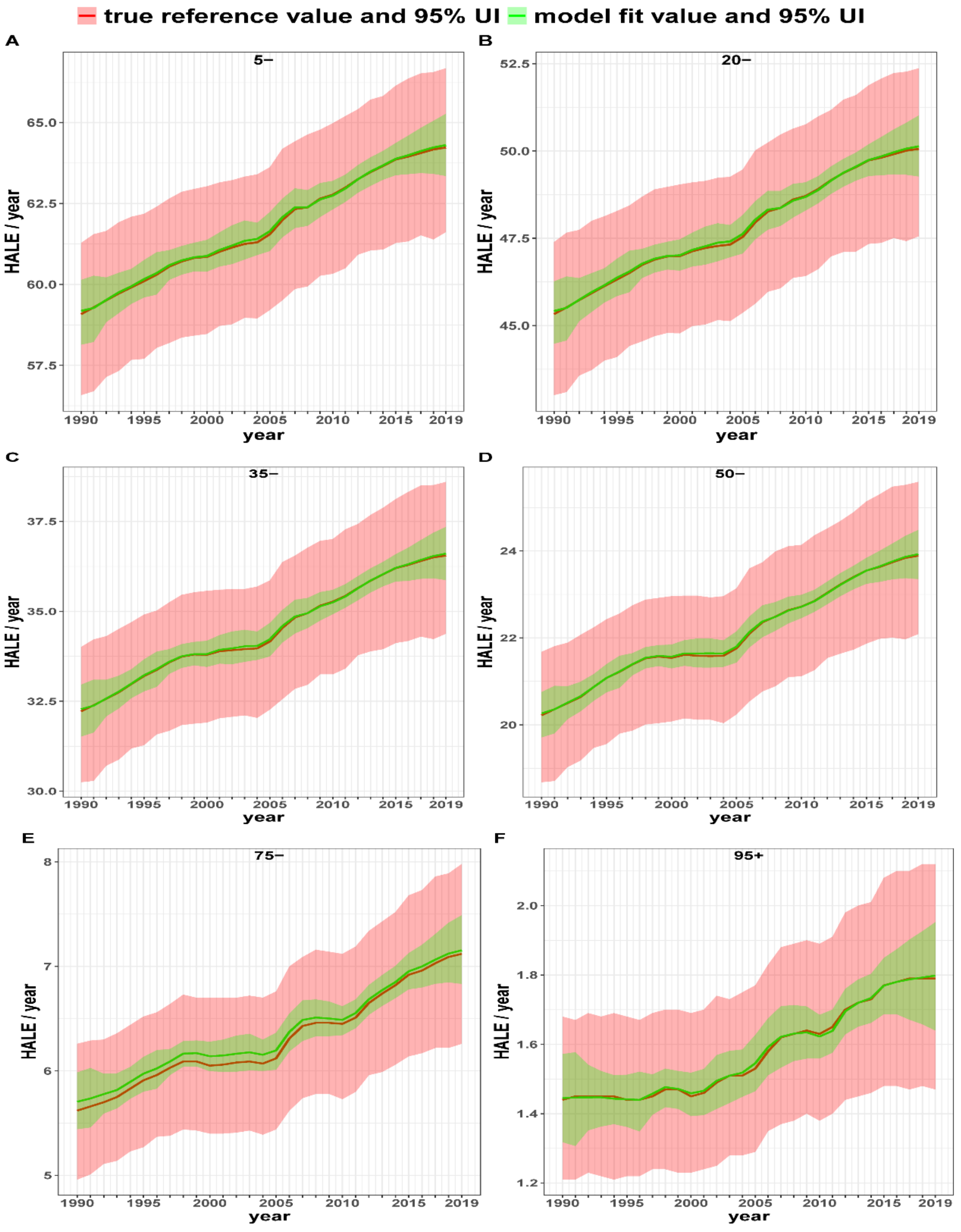

Figure S5 Comparison of model-fitted values and true reference values of HALE for different age groups, 1990-2019 (3 Variables, Both)

(A), (B), (C), (D), (E), (F) indicate comparisons of model-fitted values and true reference values of the combined HALE for the 5-9 age group, 20-24 age group, 35-39 age group, 50-54 age group, 75-79 age group and 95+ age group separately. Shaded areas indicate the corresponding 95% uncertainty intervals.
